# Scoping review: longitudinal effects of the COVID-19 pandemic on child and adolescent mental health

**DOI:** 10.1007/s00787-023-02206-8

**Published:** 2023-04-21

**Authors:** Kristin Wolf, Julian Schmitz

**Affiliations:** Department of Clinical Child and Adolescent Psychology, Wilhelm-Wundt-Institute for Psychology, University of Leipzig, Leipzig, Germany

**Keywords:** COVID-19 pandemic, Coronavirus, Childhood and adolescence, Mental health, Longitudinal, Scoping review

## Abstract

The COVID-19 pandemic and associated containment measures have massively changed the daily lives of billions of children and adolescents worldwide. To investigate the global longitudinal effects on various mental health outcomes over a period of 1.5 years, we conducted a scoping review in accordance with the guidelines of the Preferred Reporting Items for Systematic Reviews and Meta-Analyses extension for Scoping Reviews (PRISMA-ScR). We included the peer-reviewed articles from PubMed, Web of Science, and APA PsycInfo that were published between December 2019 and December 2021, followed a longitudinal or repeated cross-sectional design, and quantitatively assessed with clinical questionnaires the effect of the COVID-19 pandemic or a related stressor on mental health indicators in community samples of children and adolescents.

The results of our qualitative analysis of 69 studies indicate a general trend of less psychological well-being and more mental health problems, such as heightened stress, and depressive and anxiety symptoms during the pandemic. Data suggest that both protection measure intensity and infection dynamics were positively associated with severity of the psychopathology. The most reported influencing factors were age, gender, socio-economic status, previous state of mental and physical health, self-regulation abilities, parental mental health, parenting quality, family functioning, social support, isolation and loneliness, health-related worries, and consistent routines and structure. Our results demonstrate that children and adolescents worldwide have experienced more mental health problems due to the COVID-19 pandemic. They call for improved access to child and adolescent mental health care and prioritisation of child and adolescent welfare in political decision making.

**Supplementary Information:**

The online version contains supplementary material available at 10.1007/s00787-023-02206-8.

## Introduction

### The COVID-19 pandemic

In December 2019, the outbreak of a respiratory disease caused by the novel virus “Severe Acute Respiratory Syndrome Coronavirus type 2” (SARS-CoV-2) was reported in the People’s Republic of China [1]. The outbreak rapidly developed into an epidemic in that country by January 2020 and quickly spread to other regions of the world. On  11 March 2020, The World Health Organization (WHO) declared the spread of the virus-induced disease, COVID-19, a global pandemic [1]. By the end of February 2023, more than 757 million confirmed cases of COVID-19, over 6.85 million of which were fatal [2], were reported to the WHO. Further, it was estimated that in the years 2020 and 2021, 14.83 to 14.9 million excess deaths were associated with the pandemic [3, 4]. As a result, to contain the spread of the virus, drastic measures were taken worldwide, which led to massive restrictions on everyday life and the global economic crisis of 2020 and 2021.

### Effects of the COVID-19 pandemic on the daily life of children and adolescents

For many children and adolescents, especially in high-income countries, the still ongoing pandemic represents the first confrontation with a threat of severe disease, potential death, and grief in their lives [5–7]. In many, the pandemic has caused anxiety and worries about infection and the health of themselves and of their family and friends [6, 8]. Feelings of safety, trust, and security have been replaced by the perception that the world is a dangerous and unsafe scary place [5, 9, 10]. Young people in a British survey conducted by Youngminds [10] reported loneliness and isolation, concerns about school work, and breakdown of routines to be the most stressful experiences during the pandemic.

The global economic crisis caused by pandemic-related restrictions has led to a sharp rise in unemployment and poverty around the world [11, 12]. Especially in low-income countries, these economic hardships have forced many children into exploitative and dangerous work to support their families [11]. Further, since the start of the pandemic, essential physical and mental health services have been disrupted in over 90% of countries worldwide [13, 14]. Child and adolescent access to routine immunizations and examinations has, therefore, been impaired, leading to increased morbidity and mortality [12, 13]. In the first months of the pandemic, critical mental health services were halted worldwide, which has led to massively reduced access to psychological, psychiatric, and psychosomatic in- and out-patient services [14–16]. The work of child protection services has been impaired in at least 104 countries, making it hard to prevent, report, detect, and respond appropriately to cases of child maltreatment [17, 18].

One of the biggest and most far-reaching changes in the lives of children and adolescents during the pandemic has been the closure schools and childcare facilities. These closures have been globally regarded as necessary due to the impracticability of distancing practices in school because of limited space, frequent and varied interactions among large groups of pupils, and difficulties for children (especially younger ones) to follow hygiene and distancing guidelines [19]. According to the WHO [13], school closures have led to the largest disruption of education systems in history, affecting nearly 1.6 billion students in more than 190 countries.

It is likely that closer social relationships of children and adolescents have suffered during the pandemic due to limited and discouraged socialising and imposed isolation from friends and extended family members [17, 20]. Entire family systems have experienced disrupted daily activities and pandemic-related stress. As a result, inner-familiar tension has increased and family dynamics in many households have changed [9, 12].

Taken together, the COVID-19 pandemic and the related containment measures have massively changed the daily lives of children and adolescents. For many, normal development has been impaired and stress and strain have increased while the availability of many coping, support, and protection resources has been limited.

### Effects of disasters on mental health

The unpredictability, community impact, fatalities, and persistent effects of the COVID-19 pandemic make it a disastrous event according to literature on the mental health effects of disasters [15, 21–23]. Exposure to a disastrous event requires adaption from every individual involved [24]. This adjustment process is influenced by various factors from before (e.g., mental health history), during (e.g., injury, life threat, cumulative risk exposure), and after the event (e.g., coping behaviour, family functioning) that depend on individual experiences and resources [21, 22, 25–30]. Therefore, disastrous events can produce multiple patterns of outcome (e.g., stress-resistance, transient distress with healthy adaption, breakdown without recovery, post-traumatic growth) [21, 22, 26]. This is in line with the transactional stress model by Lazarus and Folkman [31] and classic vulnerability-stress theories, which claim that the effects of negative life events on mental health depend on individual vulnerability factors, such as how an individual perceives, attends to, appraises, interprets, copes with, and remembers such events [32, 33]. Thus, the effects of exposure to the COVID-19 pandemic, a disastrous event, seem to depend on a complex interaction of individual and environmental factors.

In general, disasters have been associated with an increase in a variety of mental health problems (e.g., depression, anxiety, post-traumatic stress, acute stress reactions, adjustment disorders, substance abuse, somatic complaints, and prolonged grief) [21, 22, 25, 28]. These mental health effects can be long-lasting, sometimes found even 12–20 years after a disastrous event [25, 28, 30].

However, catastrophes typically studied in disaster research are restricted to relatively short one-time events with ultimate and intense consequences in a particular area (e.g., earthquakes, hurricanes, oil spills, and terrorist attacks). In contrast, the COVID-19 pandemic is a global and long-lasting emergency state with stress factors of changing intensities and differential effects unfolding over time. There is still a lack of theoretical models on both the effects of this kind of disaster on mental health and on factors that make individuals more vulnerable to suffer negative mental health consequences.

### Research on the effect of the COVID-19 pandemic on child and adolescent mental health

Since the COVID-19 pandemic began in spring 2020, a lot of research into its mental health consequences has been conducted. Some reviews and meta-analyses have already tried to summarise this enormous body of research. For instance, Santomauro et al. [34] estimated in their systematic review that there has been an increase of 27.6% in depression and of 25.6% in anxiety disorders in the general population due to the pandemic. In addition, a number of reviews and meta-analyses which assessed the psychological consequences of the COVID-19 pandemic on different age groups have found a strong increase of mental health problems in children and adolescents [9, 35–41].

These previous reviews and meta-analyses have included mostly cross-sectional studies. This makes it difficult to distinguish between pre-existing levels of psychopathology and the pandemic-specific consequences and can lead to an overestimation of effects.

Longitudinal studies are more appropriate for investigating these effects because they allow a direct detection of developmental pathways across time. Unfortunately, the few meta-analyses that use longitudinal assessments of mental health symptoms focus on the general population and not on children and adolescents in particular [42–44].

### The current review

The current study fills in some gaps by reviewing only longitudinal and repeated cross-sectional studies on the mental health consequences of the COVID-19 pandemic on child and adolescent mental health that have been published over a course of two years. The objectives are to assess the pandemic’s effects on a broad spectrum of mental health outcomes, to learn about factors influencing these effects, to investigate long-term psychological consequences over the course of the pandemic, and to create a large sample of studies conducted in many different countries to obtain a higher generalisability of the findings.

This study might also be beneficial to disaster theory and research because the COVID-19 pandemic significantly differs from disasters frequently studied in terms of duration, intensity, and differential effects. Previously studied disasters include, for example, natural catastrophes (e.g., hurricanes, oil spills, floods), human-made technological disasters (e.g., the nuclear accident at Chernobyl), acts of mass violence and terrorism (e.g., the attack on the World Trade Center in 2001), and previous epidemics and pandemics (e.g., the H1N1 influenza (swine flu) pandemic) [21, 25–27, 30, 45].

The following research questions were investigated in this paper:

1. How did the prevalence of mental health problems in the general population of children and adolescents change from before the COVID-19 pandemic to during the pandemic?
2. How did the prevalence of mental health problems in the general population of children and adolescents develop over the course of the pandemic?
3. What are factors that influence the potential effects of the COVID-19 pandemic on child and adolescent mental health?

Mental health can be measured by both broader outcomes (i.e., quality of life, well-being, life satisfaction, psychological stress, and affect) and by specific symptoms of common mental health disorders in childhood and adolescence. These involve internalising symptoms (including depression and anxiety), externalising symptoms (including hyperactivity, inattention, and disruptive behaviour problems), psychosomatic complaints, addictive behaviour (including substance abuse and excessive use of electronic media), and post-traumatic stress symptoms. These symptom categories match commonly described outcomes in research discussed previously and cover a broad range of mental health problems that can occur in childhood and adolescence.

## Methods

### Literature research

This review is in accordance with the guidelines proposed by the Preferred Reporting Items for Systematic Reviews and Meta-Analyses extension for Scoping Reviews (PRISMA-ScR) [46]. There is no registered protocol. The literature search was conducted in the PubMed, Web of Science, and APA PsycInfo databases, each consulted last on 12 January 2022. The following was our search strategy to identify studies that examined the effect of the COVID-19 pandemic on child and adolescent mental health: “(COVID-19 OR corona* OR SARS-CoV-2 OR novel coronavirus) AND (psychological OR psychopatholog* OR mental health OR psychiatric disorders OR interna* OR externa* OR depress* OR anxi* OR sleep* OR hyperact* OR ADHD OR attention OR autis* OR neurodevelopment* OR disruptiv* OR conduct OR defiant OR oppositional OR emotional OR behavio* OR addict*) AND (adolescent* OR child* OR youth OR minor* OR teenager* OR juvenile*)”. To ensure sufficient data, we initially included a broad range of mental health symptoms in the search strategy. The terms used were based on the strategy used in a published review on child and adolescent psychopathology in the context of social competence [47].

If available at the respective databases, we used automatic filters as follows: The search terms named above had to appear in the title and/or abstract of the studies, the studies had to be in peer-reviewed journal articles written in the English language and published between December 2019 and December 2021, the participants had to be aged between 0 and 18 years, and the methodology was restricted to quantitative designs.

We identified additional studies via the ancestry approach by examining reference lists of studies included in this review and reviews and meta-analyses on the same topic. The first author manually screened the identified studies multiple times based on titles, abstracts, and methodology (i.e., longitudinal, repeated, or one-time cross-sectional). Eligible studies were then textually reviewed, and the results were summarised in a table. The second author confirmed the literature search and inclusion process. This research procedure is equivalent to other systematic reviews (e.g., [47]).

We filtered the results further using the following inclusion criteria: The research question should focus on the effect of the COVID-19 pandemic (i.e., by comparing measurements prior to and during the pandemic) or a pandemic-related stressor (e.g., confinement, quarantine) on a mental health outcome. Mental health could be assessed via global measures of psychological distress, quality of life, life satisfaction, affect, and well-being or via measures of specific symptoms of common mental disorders in childhood and adolescence.

Due to the large body of research found, we placed a closer focus on the following symptom groups: internalising symptoms, including depression, anxiety, and post-traumatic stress disorder; externalising symptoms, including hyperactivity, inattention, and disruptive behaviour problems; psychosomatic complaints; and addictive disorders such as substance abuse or excessive use of electronic media.

Because changes over time are more directly detectable in longitudinal research and the general population of children and adolescents is best represented in community samples, we decided to include only studies with a longitudinal or repeated cross-sectional research design with at least one measurement during the pandemic and only studies that used community samples when it became clear that enough of such studies were available. Because most identified studies assessed mental health outcomes via self- or others-report questionnaires, we also decided to include only studies that used this methodological approach to gain higher homogeneity and thus better comparability among studies.

Exclusion criteria were cross-sectional research designs with a one-point assessment of mental health, qualitative assessments of mental health outcomes, clinical sample use (i.e., children and adolescents with pre-existing mental or physical health disorders), or specific sub-sample use (e.g., young athletes or LGBTQ+ youth). Similarly, studies with a predictor only loosely connected to the COVID-19 pandemic (e.g., use of electronic media) and/or an outcome not focussed on mental health (e.g., family functioning, academic learning, or lifestyle changes) met the exclusion criteria. Research assessing intervention or prevention programmes, or diagnostic instruments was also excluded.

**Fig. 1:**
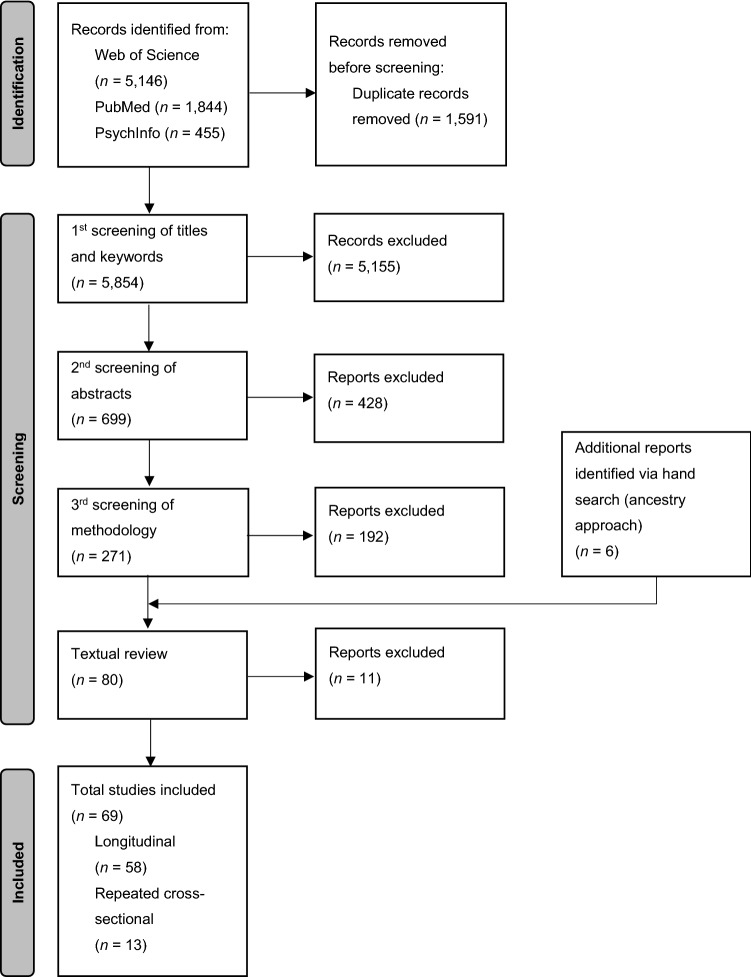
Process and results of literature research. Adapted from Page et al. 2021 [48]

The process of study selection is depicted in Fig. 1.

### Synthesis of results

To synthesise the body of research that supports changes in distinct mental health outcomes more explicitly, we sorted the findings of studies comparing measures before and during the pandemic by examined outcome (e.g., depressive symptoms) and reported change in outcome (i.e., increase, no change, or decrease from before to during the pandemic). We calculated and tabulated the number of studies that examined each mental health indicator and the number of findings for each possible direction of change.

Wherever possible, we grouped together the outcomes that measured overlapping constructs and symptom categories. The larger pools of studies for each indicator allow for more profound conclusions. We summarised mental health indicators from outcomes as follows:

- “Psychological well-being” and “quality of life” were grouped together (c.f., [49]).
- Total scores of scales assessing multiple mental health outcomes were summarised as “global measures of mental health problems”.
- “Internalising symptoms/problems” and “emotional symptoms/problems” were summarised as “internalising symptoms” (c.f., [50, 51]).
- “Externalising symptoms/problems” and “behavioural symptoms/problems” were summarised as “externalising symptoms” (c.f., [50, 51]).
- “Conduct”, “oppositional”, “challenging”, and “impulsive behaviour symptoms” were summarised as “disruptive behaviour symptoms” (c.f., [52]).
- “Inattention”, “attentional problems”, and “sluggish cognitive tempo” were summarised as “cognitive symptoms”.

In addition to those above, we included the following psychopathological indicators and symptoms because they appeared in studies chosen for this review: resilience, suicidal symptoms, neurotic symptoms, dissociative symptoms, eating disorder symptoms, and psychotic symptoms.

To examine the overall directions of changes in mental health, we added the numbers of findings that supported each of increase, no change, and decrease in a mental health indicator. We grouped the outcomes as “indicators of good mental health” if they were positively associated with mental health (e.g., well-being, quality of life, life satisfaction) and “indicators of mental health problems” if they were negatively associated with mental health (e.g., stress, negative affect, psychopathological symptoms). We synthesised the results of studies of changes over the course of the pandemic separately. The emphasis was thereby on findings on the effects of certain health protection measures (i.e., lockdown, confinement) and the general severity of restrictions on mental health outcomes.

To examine the factors that influence the effect of the pandemic on mental health (i.e., moderators, mediators, correlates), we collected those that were reported in the studies as “COVID-19 related stressors” or “other variables” then tabularly sorted them into the following factor categories:

- Sociodemographic
- Intra-individual
- Parental/family
- Social
- COVID-19-related
- Behavioural

We used two additional approaches post-hoc to review the studies’ findings and identify sources of variance among outcomes. We sorted the studies that examined changes from before to during the pandemic by both age of the participants (<  six years, six–12 years, > 12 years) and country where the study was conducted to investigate systematic effects. To evaluate age effects, we counted findings of an increase, no change, or decrease in each mental health indicator separately. To evaluate the influence of region, we sorted findings by direction of change in mental health and not by specific indicators due to the small number of studies for each country.

## Results

### Sample characteristics

In total, we included 69 studies, published between September 2020 and December 2021 that assessed around 130,000 participants in this review. Fifty-eight of the studies used a longitudinal, within-subjects design. Thirteen studies applied a repeated cross-sectional, between-subjects design (e.g., cohort designs, matched convenience samples). Two studies analysed both within-subjects (i.e., intra-individual development) and between-subjects comparisons (i.e., age group comparisons) [53, 54].

All included studies conducted their in-pandemic assessment between February 2020 and June 2021, which covers a period of 17 months. Most were carried out in the months of April (*n* = 33), May (*n* = 37), and June 2020 (*n* = 29), which correlates with the first wave of the pandemic and the first lockdown in many countries. Far fewer studies were conducted after the summer of 2020 and in the first half of 2021. In 45 of the 69 included studies, data were collected during lockdown, confinement, or stay-at-home orders. Fourteen studies assessed children’s and adolescents’ mental health indicators and/or symptoms multiple times during the pandemic [53–66].

In total, our review included data from 21 countries, comprising 14 European, four Asian, two North American countries, and one South American country. Most studies were conducted in the United States of America (*n* = 14), China (*n* = 8), the United Kingdom (*n* = 7), Germany (*n* = 6), and Canada (*n* = 6).

All age groups between three and 18 years were included in this review. Early teenage years (i.e., 12–14 years) appeared most frequently in the study population, while research investigating effects on early childhood was rather rare. More concretely, nine studies included participants aged six years or younger, 34 studies included participants aged six–12 years, and 44 studies included participants aged above 12 years. Note that some studies fell into multiple groups.

### Overview of included studies

Table 1 displays the 58 longitudinal studies and the two studies using both longitudinal and repeated cross-sectional approaches. Table 2 depicts the remaining 11 repeated cross-sectional studies.

**Table 1:** Overview of longitudinal studies (Same Sample)

No	Authors	Assessment during pandemic	Country	*M*_Age_/Age range	*N*	Waves of assessment	COVID-19-related stressors/Other variables	Assessment of children’s and adolescents’ mental health	Main findings
1	Achterberg et al. [67]	04–05/2020 (lockdown)	Netherlands	*M* = 12.00 years 10–13 years	106 parents and 151 children	T1: 2016 T2: 2017 T3: 2018 T4: 2019 T5: 04–05/2020	Parents’ over-reactivity Children’s and parents’ positive and negative coping strategies Well-being of parents Children’s perceived stress during lockdown	Parent-report Internalising and externalising behaviour via Strengths and Difficulties Questionnaire (SDQ)	No change in internalising symptoms from T1–4 to T5 Stop of gradual decrease externalising behaviour from T2 to T4 at T5 Mediator of change of externalising problems: perceived stress (predicted by negative coping strategies of children and parents, prior parental over-reactivity)
2	Aleksandrov and Okhrimenko [68]	05/2020 (after two months of quarantine)	Ukraine	15–17 years	152 adolescents	T1: 11/2019 T2: 05/2020		Self-report Neurotic disorders via Neuroticism Questionnaire Neurotic personality factors via Multifactor Questionnaire	Increase of risk of neurotic disorders neurasthenia, psychasthenia, hypochondria, and depression from T1 to T2 No change in symptoms of autonomic disturbances, histrionic personality disorder, derealisation, and depersonalization from T1 to T2 Correlates of increased neurotic symptoms: isolation, emotional instability, anxiety, timidity, worry, tension
3	Alt et al. [69]	05–07/2020 (lockdown)	Germany	*M*_T1_ = 16.11 years 14–17 years	843 adolescents	T1: 10/2018–08/2019 T2: 05–07/2020	Extraversion Loneliness	Self-report Depression via State-Trait Depression Scales (STDS)	Increase in depressive symptoms and loneliness from T1 to T2 Highly extroverted adolescent experienced larger rise in depressiveness, mediated through increase in loneliness
4	Bélanger et al. [70]	05–07/2020 (lockdown)	Canada	*M*_T1_ = 14.1 years Grades 9–12 ~ 13–18 years	2099 adolescents	T1: 2018 T2: 2019 T3: 05–07/2020		Self-report Psychosocial well-being via Flourishing Scale (FS) Depression symptoms via Center for Epidemiologic Studies Depression Scale Revised (CESDR-10) Anxiety symptoms via Generalized Anxiety Disorder Scale (GAD-7)	Increase in depression and anxiety, decrease in psychological well-being across all waves Changes between T2 und T3 were not greater than between T1 and T2, i.e., no deterioration of mental health due to initial COVID-19 response Sex and age were no significant moderators of symptom development
5	Bernasco et al. [71]	04–07/2020 (partial lockdown and school closure)	Netherlands	*M*_T1_ = 11.6 years	245 children and their parents	T1: 2019 T2: 04–07/2020	Friend support Time spent with friends COVID-19 related stress	Parent-report Internalising symptoms via internalising problems scale of the Child Behavior Checklist (CBCL) self-report Anxiety and depression symptoms via Revised Child Anxiety and Depression Scale (RCADS)	Decrease from T1 to T2 in self-reported depression, anxiety, and friend support No change in parent-reported internalising symptoms Higher pre-COVID-19 friend support predicted less self- and parent-reported internalising problems during COVID-19 Time spent with friends and COVID-19 related stress were no significant moderators
6	Bignardi et al. [72]	04–06/2020 (before and during lockdown)	United Kingdom	*M* = 9.4/10.5 years 7.6–11.6 years	168 children and their caregivers and teachers	T1: 06–12/2018 and 12/2018–09/2019 T2: 04–06/2020		Caregiver- and teacher-report Emotional symptoms via Strengths and Difficulties Questionnaire (SDQ) caregiver- and self-report Depression and anxiety via Revised Child Anxiety and Depression Scale (RCADS-short form)	Medium to large increase in depression symptoms from T1 to T2 No changes in anxiety and emotional problems from T1 to T2
7	Borbás et al. [66]	03–06/2020 (lockdown and partial school closure)	Switzerland	*M*_T1_ = 9.58 years *M*_T2_ = 10.69 years 7–17 years	26 children and 21 mothers	T1: 03/2018–02/2020 T2-T8: 03–05/2020 (bi-weekly assessments)	Mothers’ burden of care Mothers’ well-being (anxiety, depression, physical health, distress) Time spent outside Meeting with friends	Parent-report Child behaviour via Child Behavior Checklist (CBCL) self-report Emotional symptoms, conduct problems, peer problems, hyperactivity and prosocial behaviour via Strengths and Difficulties Questionnaire (SDQ) Subjective mood ratings	No change in children’s average behavioural and emotional problems from T1 to T2–8 Decrease in conduct problems, hyperactivity, peer problems, emotional, and behavioural problems from T2 to T8, i.e., decrease in symptoms during restrictions Increase in prosocial behaviour from T2 to T8 Significant predictors of children’s symptoms and mood: mothers’ subjective burden of care, mothers’ depression, and meeting friends
8	Breaux et al. [55]	05–06/2020 (lockdown) and 07–08/2020 (lift of restrictions)	United States of America	8th grade students ~ 13 years	120 adolescents (+ 118 adolescents with ADHD not reported here)	T1: 09/2018–02/2020 T2: 05–06/2020 T3: 07–08/2020	Emotion regulation abilities	Self-report Sluggish cognitive tempo via Child Concentration Inventory, Second Edition (CCI-2) Anxiety and depression symptoms via Revised Anxiety and Depression Scales (RCADS) parent-report Hyperactivity, impulsivity, inattention, and oppositionality/defiance via Vanderbilt ADHD Diagnostic Rating Scale (VADRS)	Increase in depression, anxiety, slower cognitive tempo, inattention, and oppositional symptoms from T1 to T2 No change in hyperactivity and impulsivity across T1–T3 Decrease in all elevated symptoms (except inattention) from T2 to T3, i.e., decrease in symptoms after lift of restrictions Risk factors for increased internalising and externalising symptoms: poorer pre-COVID-19 emotional regulation abilities, lower family income (inattention), higher family income (oppositional/defiant symptoms)
9	Browne et al. [73]	03/2020 (beginning of the pandemic, prior lockdown)	Canada	*M* = 5.69 years 3–12 years	Educators of 231 children	T1: 12/2019–01/2020 T2: 02/2020 T3: 03/2020		Educator-report Mental health problems via Strengths and Difficulties Questionnaire (SDQ) Children’s impairment (e.g., relationships with teacher and classmates, academic progress, self-esteem) via Impairment Rating Scale (IRS)	Male children showed a modest decline in mental health problems (i.e., total difficulty and impairment score) at T1 to T2, i.e., mental health problems improved prior to the pandemic Worsening of mental health of male children from T2 to T3 No changes over time observed for female children
10	Chaffee et al. [74]	03–09/2020 (lockdown, stay-at-home order)	United States of America	9th-10th grade students ~ 14–15 years	1006 adolescents (T2 for 521 before stay-at-home order, for 485 after)	T1: 03/2019–02/2020 T2: 09/2019–09/2020	Physical activity	Self-report Substance use (e.g., alcohol, cigarettes, cannabis) via indication if and how often used in the last 30 days	No change in use of tobacco, cannabis, and alcohol from T1 to T2 Decrease in use of e-cigarettes from T1 to T2 (both before and after stay-at-home order) Sharp decrease in physical activity from T1 to T2 (after stay-at-home order)
11	I. H. Chen et al. [75]	03/2020 (closure of schools)	China	*M*_T1_ = 10.32 years Primary school students	535 children	T1: 10–11/2019 T2: 03/2020	Perceived academic performance Time spent on gaming Social media use Smartphone use	Self-report Problematic Internet-related behaviours via Smartphone Application-Based Addiction Scale (SABAS), Bergen Social Media Addiction Scale (BSMAS), and Internet Gaming Disorder Scale-Short Form (IGDS9-SF) Psychological distress via Depression, Anxiety, Stress Scale-21 (DASS-21)	Increase in time spent on smartphone and social media use, not in time spent on gaming from T1 to T2 Decrease of problematic social media use and problematic gaming from T1 to T2 Increase in psychological distress from T1 to T2 Illness status and problematic Internet-related activities predicted psychological distress more strongly at T2 than at T1
12	Y. Chen et al. [76]	02–11/2020	Sweden	*M*_T1_ = 13.6 years ~ 15 years at T2	1900 adolescents (T2 for 1316 before 02/2022, for 584 after)	T1: 09/2015– 06/2019 T2: 09/2017–01/2020 control group 02–11/2020 COVID-19 exposed group	Relationships with parents and peers Social support and relationships to peers School environment Health behaviours (sleep, physical activity)	Self-report Stress via 10-item Perceived Stress Scale (PSS-10) Psychosomatic symptoms via the PsychoSomatic Problem Scale (PSP Scale) Psychological well-being via Oxford happiness questionnaire (OHQ)	Increase of stress, psychosomatic symptoms and decrease of happiness from T1 to T2 for entire sample No significant difference between control and exposed group at T2 No significant change in relationships to peers and parents, sleep duration and physical activity from T1 to T2
13	Cohen et al. [77]	05–06/2020 (reopening phase after lockdown)	United States of America	*M* = 14.53–15.22 years	24 adolescents (15 healthy, 9 with early life stress experience)	T1: 08/2019–02/2020 T2: 05–06/ 2020	Early life stress Parent and peer relationship quality Coping with COVID-19 pandemic	Self-report Symptoms of depression and anxiety via Patient-Reported Outcomes Measurement Information System (PROMIS)	Increase in symptoms of depression and anxiety from T1 to T2 only for healthy adolescents but not for early life stress exposed adolescents Greater negative and fewer positive emotions in participants with early life stress than healthy participants Protective factors: coping by talking with friends, prioritizing sleep
14	Daniunaite et al. [78]	09–10/2020 (partial reopening of schools)	Lithuania	*M*_T1_ = 13.87 years 12–16 years at T1	331 adolescents	T1: 03–06/2019 T2: 10/2020		Self-report Emotional symptoms, conduct problems, peer problems, hyperactivity and prosocial behaviour via Strengths and Difficulties Questionnaire (SDQ)	Increase in hyperactivity/inattention, emotional symptoms, and prosocial behaviour from T1 to T2 No change in conduct or peer problems from T1 to T2 Significant increase in psychosocial problems in 70.7% of participants Increase only in peer problems in 19.6% No negative change in 9.7%
15	de France et al. [79]	05–06/2020 (start of loosening of restrictions)	Canada	*M*_T1_ = 13.9 years *M*_T5_ = 16.21 years	136 adolescents	T1–4: 2018–2020 T5: 05–06/2020, six months after T4	Impact of the pandemic (health, financial, lifestyle, fear of coronavirus) Emotion dysregulation	Self-report nxiety via Multidimensional Anxiety Scale for Children (MASC) Depression via Children’s Depression Inventory (CDI)	Higher anxiety and depression scores at T5 than predicted by previous trajectories Level of perceived lifestyle impact due to the pandemic associated with deviations from personal trajectories (moderator)
16	Deng et al. [80]	03–06/2020 (lockdown)	United States of America	*M*_T1_ = 11.77 years *M*_T2_ = 12.64 years 9–15 years	115 children and adolescents	21/28-day daily diary period T1: 01–09/2019 T2: 03–06/2020	pre-existing emotion regulation strategies COVID-19 related worries and isolation	Self-report Positive and negative affect level via 10-item Positive and Negative Affect Schedule for Children (PANAS-C)	No significant difference in the mean levels of positive affect between T1 and T2 Increase in levels of negative affect from T1 to T2 No change in variability of negative affect but decrease in variability of positive affect from T1 to T2 Positive emotion regulation strategies as predictors of more positive affect during pandemic Negative emotion regulation strategies correlating with COVID-19 related worries as predictors of more negative affect during pandemic
17	Essler et al. [56]	04–05/2020 (lockdown) and 07/2020 (loosening of restrictions)	Germany	3–10 years	890 parents	T1: 04–05/2020 T2: 07/2020	Parental strain Parental self-efficacy Parent–child relationship quality	Parent-report Well-being via KIDSCREEN-52 Health-Related Quality of Life Questionnaire for Children and Adolescents (12 items chosen for this study) Problem behaviour via Strengths and Difficulties Questionnaire (SDQ)	Decreases in parental stress, children’s problem behaviour and increase in children’s well-being from T1 to T2, i.e., decrease in symptoms after lift of restrictions Decreases in parental strain predicted increase in child well-being and decrease in child problem behaviour from T1 to T2 Children’s problem behaviour at T1 predicted parental stress at T2 and parental stress at T1 predicted children’s problem behaviour at T2
18	Ezpeleta et al. [81]	06/2020 (after lockdown)	Spain	*M*_T2_ = 13.9 years 12 years at T1 13 years at T2	197 parents	T1: 2019 T2: 06/2020	Adolescents’ living conditions during lockdown (physical environment, relationships, activities, feelings, and behaviours)	Parent-report Emotional symptoms, conduct problems, peer problems, hyperactivity and prosocial behaviour via Strengths and Difficulties Questionnaire (SDQ)	Increase in conduct, peer, prosocial and total problem scores from T1 to T2 Decrease in emotional problems from T1 to T2 No change in hyperactivity/inattention problems from T1 to T2 Factors associated with elevated symptoms: unhealthy activities, feelings, and behaviour (e.g., frustration, boredom, sleep problems, excessive concerns about health), worsening of relationships with others, dysfunctional parenting style
19	Feinberg et al. [82]	04–05/2020 (lockdown)	United States of America	*M*_T1_ = 7 years *M*_T2_ = 9.9 years	206 parents from 129 families	T1: 2017–2019 T2: 04–05/2020	Parents’ depression and anxiety Household income Parenting quality	Parent-report Externalising and internalising problems via Strengths and Difficulties Questionnaire (SDQ)	Increase in children’s internalising and externalising problems from T1 to T2 Increase in parents’ depression and anxiety, decrease in co-parenting and parenting quality from T1 to T2 Risk factor: lower family income
20	Fung et al. [57]	03/2020 and 06/2020 (outbreak to post-lockdown)	China	*M*_T3_ = 11.6 years 9.8–13.68 years at T1	489 children and adolescents	T1: 01/2020 T2: 03/2020 T3: 06/2020	Perceived weight stigma	Self-report Problematic smart phone use via Smartphone Application-Based Addiction Scale (SABAS) Problematic social media use via Bergen Social Media Addiction Scale (BSMAS) Psychological distress, depression and anxiety via Depression, Anxiety, Stress Scale-21 (DASS-21)	No change of depressive symptoms from T1 to T2, decrease of depressive symptoms from T2 to T3, i.e., decrease of symptoms from outbreak to post-lockdown Decrease of anxiety and weight stigma from T1 to T2, no change from T2 to T3, i.e., decrease of symptoms from pre-pandemic to outbreak No change of depressive and anxiety symptoms from T1 to T3 Increase of stress and problematic smartphone use from T1 to T2 and T3, i.e., increase of symptoms from pre-pandemic to outbreak and post-lockdown Increase in association between problematic social media use and depression/anxiety from T1 to T3
21	Genta et al. [83]	06/2020 (lockdown)	Brazil	*M*_T1_ = 15.0 years *M*_T2_ = 16.4 years	94 adolescents	T1: 03/2019 T2: 06/2020	Sleep quality Sleepiness Chronotype	Self-report Quality of life via World Health Organization Quality of Life Questionnaire-abbreviated version (WHOQOL-BREF)	Worsening of physical and psychological domains of quality of life from T1 to T2 Improvement of environmental domain of quality of life from T1 to T2 Increase of sleep duration and sleep quality only among more students who were sleep deprived pre-pandemic Delayed wake-up times and shifted chronotype to “eveningness” during pandemic
22	Giménez-Dasí et al. [84]	04/2020 (lockdown)	Spain	*M* = 7.02 years 3.2–11.1 years	113 parents	T1: 02/2020 T2: 04/2020		Parent-report Attentional problems, depression, challenging behaviours, emotional regulation, hyperactivity, and willingness to study via System of Evaluation of Children and Adolescents (SENA)	Increase in emotional, attentional, behavioural (hyperactivity, impulsivity) problems, and decrease in willingness to study among six- to 10-year-olds from T1 to T2 No change among three-year-old children No changes in depression and challenging behaviour
23	Giménez-Dasí et al. [58]	03–04/2020 (lockdown) and 12/2020–02/2021	Spain	*M* = 8.53 years 6–11 years	215 children	T1: 12/2019– 02/2020 T2: 03–04/2020 T3: 12/2020–02/2021		Self-report Anxiety via anxiety scale from System of Evaluation of Children and Adolescents (SENA)	Decrease in anxiety from T1 to T2 and T3, only in children between eight and 11 years No change in anxiety from T1 to T2 for whole sample No change over all waves for children younger than 8 years
24	Gladstone et al. [85]	05/2020 (lockdown)	United States of America	*M* = 14.5 years 12–18 years	228 adolescents	T1: 11/2019– 01/2020 T2: 05/2020	Vulnerability (cognitive styles, attitudes) Protective factors (resilience, conflict behaviour) COVID-19 related distress	Self-report Depressive symptoms via Patient Health Questionnaire-Adolescent (PHQ-A)	Increase in depressive symptoms from T1 to T2 (4.9% to 5.7% with moderately severe to severe symptoms) Overall mean of depressive symptoms in minimum symptom range at T1 and T2 Predictors of increase in depressive symptoms: low resilience, negative cognitive style, COVID-19-related distress, female gender
25	Hafstad et al. [86]	06/2020 (reopening of schools)	Norway	12–16 years	3572 adolescents	T1: 02/2019 T2: 06/2020	Pandemic-related worries Loneliness Family affluence	Self-report Anxiety and depressive symptoms via Hopkins Symptom Checklist (HSCL-10)	Slight increase of anxiety and depression from T1 to T2 (5.5% to 6.3%) caused by increase in age from T1 to T2 Predictors of higher levels of symptoms: female gender, pre-existing mental health problems, living in a single-parent-household Predictors of lower increase in symptoms: low family socio-economic status, history of maltreatment
26	Hollenstein et al. [87]	05–06/2020 (lockdown)	Canada	*M*_T1_ = 12.49 years T1: 12–13 years	155 mothers, 146 adolescents	T1: 09/2019–03/2020 T2: 05–06/2020	Mothers’ anxiety and depression Mothers’ hardship Child positive and negative changes (e.g., sleep, school pressure, time with family, worry about illness)	Self-report Anxiety symptoms via Beck Anxiety Inventory (BAI) Depressive symptoms via The Children’s Depression Inventory, Second Edition (CDI-2)	Increase in adolescents’ and mothers’ depression from T1 to T2 (especially in females) Decrease in adolescents’ and mothers’ anxiety from T1 to T2 (especially in males and for individuals with high pre-pandemic anxiety) Risk factor for stronger increase in depressive symptoms: more negative changes due to COVID-19 (e.g., less contact to friends, stress, worry about health)
27	Hu and Qian [88]	07/2020 (loosening if restrictions)	United Kingdom	*M*_T2_ = 13.3 years 10–16 years at T2	886 adolescents	T1: before 03/2020 T2: 07/2020	Family income Living conditions (e.g., co-residence with other children, single parent)	Self-report Emotional symptoms, conduct problems, peer problems, hyperactivity and prosocial behaviour via Strengths and Difficulties Questionnaire (SDQ)	Increase in psychopathological symptoms (emotional problems, conduct problems, hyperactivity, peer relationship problems) and decrease in prosocial tendencies from T1 to T2 for adolescents with relatively good mental health at T1 Decrease in psychopathological symptoms, increase in prosocial behaviour from T1 to T2 in adolescents with worse than median mental health at T1 Boys showed smaller increase in emotional problems but greater decrease in prosocial tendency than girls Risk factors for negative mental health impact: only child, single-parent, low family income
28	Hussong et al. [89]	05–07/2020 (lockdown, school closures)	United States of America	*M*_T4_ = 13.6 years 6–9 years at T1 12–16 years at T4	105 parent–child dyads	T1: 2013/14 T2: 2015/2016 T3: 2016/2017 T4: 05–07/2020	Child-reported self-efficacy Child-reported optimism Child-reported coping	Parent-report Internalising and externalising problems via Paediatric Symptom Checklist (PSC)	Increase in mental health symptoms from T1–3 to T4 Protective/mitigating factors: self-efficacy, problem-focused engaged coping Optimism did not mitigate risk of mental health problems Risk factors for worsening of mental health: emotion-focused engaged and disengaged coping
29	Hyunshik et al. [90]	10/2020	Japan	*M*_T2_ = 4.8 years 3–5 years at T2	290 parent–child dyads (high socio-economic status)	T1: 10/2019 T2: 10/2020	Physical activity Adherence to WHO-recommended screen time Prosocial behaviours Sleep duration	Parent-report Emotional symptoms, conduct problems, peer problems, hyperactivity and prosocial behaviour via Strengths and Difficulties Questionnaire (SDQ)	Increase in sedentary behaviour and hyperactivity from T1 to T2 No change in total difficulty score, emotional problems, conduct problems and peer problems fromT1 to T2 Decrease in physical activity, adherence to recommended screen use and prosocial behaviours from T1 to T2
30	Jiang et al. [91]	04/2020 (lockdown)	China	*M* = 13.85 years 11–16 years	257 students	T1: 11–12/2019 T2: 04/2020	Social support	Self-report Resilience via Resilience Scale for Chinese Adolescents (RSCA) Depression via Center for Epidemiologic Studies Depression Scale for Children (CES-DC) Sleep problems via Pittsburgh Sleep Quality Index (PSQI)	Decrease of resilience from T1 to T2 Resilience as a protective factor against depression and sleep problems Social support as a moderator of effect of resilience on depression and sleep
31	Larsen et al. [92]	04–05/2020 (school closures, social isolation)	Norway	*M* = 11.43 years	442 children and their parents (sample from family counselling centres)	T1&T2: 12/2017–07/2019 T3: 04–05/2020	Psychological vulnerability at T1/T2 Home-schooling experiences Family stress and instability Screen time use Missing friends Worry about infection	Self-report Emotional symptoms via five items created for the study Somatic/cognitive symptoms via three items created for the study Worry reactions via two items created for the study	Decrease in emotional symptoms (feelings of sadness, anger, anxiety, unsafeness) from T1 and T2 to T3 Increase in somatic/cognitive symptoms (feelings of loneliness, sleeping and concentration problems) T1 and T2 to T3 Predictors of higher psychological vulnerability: home-schooling experiences, family stress and instability, missing friends, worry about infection, older age
32	Liang et al. [59]	03–05/2020 (lockdown)	Italy	*M* = 14.13 years 11–18 years	288 parents	T1: 03/2020 T2: 04/2020 T3: 05/2020	Parental stress Parental expressive suppression (tendency to inhibit expression of emotions)	Parent-report Depression via Impact Scale of COVID-19 and Home Confinement on Children and Adolescents, and Short Mood and Feelings Questionnaire-Parent Version (SMFQ-P) Anxiety via Impact Scale of COVID-19 and Home Confinement on Children and Adolescents, and Spence Children’s Anxiety Scale-Parent Version (SCAS-P-8)	Increase in anxiety and depression symptoms from T1 to T2 Slight decrease of anxiety symptoms and no change in depression symptoms from T2 to T3 Increased restrictions associated with more severe symptoms Parental stress associated with adolescents’ internalising symptoms over parental expressive suppression
33	Liao et al. [93]	07/2020	China	*M* = 13.4 years 11–16 years	2496 adolescents	T1: 12/2019 T2: 07/2020	Sleep duration	Self-report Depression via Center of Epidemiological Studies Depression Scale for Children (CES-DC)	Increase of depression and decrease in sleep duration from T1 to T2 Shortened sleep duration at T1 predictive of increased depressive symptoms at T2, depressive symptoms at T1 predictive of decreased sleep duration at T2
34	Mastorci et al. [94]	04/2020 (lockdown)	Italy	*M* = 12.5 years 10–14 years	1289 adolescents	T1: 09–10/2019 T2: 04/2020	Dietary habits Physical activity Housing, living in a city/countryside Sleep quality Maintaining contact with friends over smartphone use	Self-report Health-related quality of life via KIDSCREEN-52	Decrease of psychological and physical well-being from T1 to T2 (reduced autonomy, worse mood, and emotion, reduced financial resources, worsening in relationships to family and friends) Moderating factors: gender, housing, and environmental conditions Females showed lower scores for psychological and physical well-being, self-perception, and mood/emotion than boys Males showed lower scores in physical activity than girls Greater reduction in autonomy and peer relationships in adolescents living in a village than in a city Greater reduction of physical well-being for adolescents living in a city and in an apartment without green space than adolescents living in a village and in a house
35	O’Kane et al. [95]	05–06/2020 (lockdown)	Ireland	*M* = 12.8 years 12–14 years	94 female adolescents	T1: 09–10/2019 T2: 05–06/2020	Emotion regulation Self-efficacy Physical activity Sleep quality Social media usage and emotional investment in social media Body weight and appearance satisfaction	Self-report Health-related quality of life via KIDSCREEN-10	Decrease in health-related quality of life and motivation to exercise from T1 to T2 Increase in happiness with appearance from T1 to T2 No change in self-reported physical activity, sleep quality, social media usage from T1 to T2
36	Orgilés et al. [60]	03–05/2020 (lockdown)	Italy, Spain, Portugal	*M* = 8.94 years 3–18 years	Parents of 624 children and adolescents	T1: 2 weeks into first lockdown 2020 T2: 5 weeks after beginning of lockdown T3: 8 weeks after beginning of lockdown	Country: Italy: first European country massively affected by the pandemic, first European lockdown Italy and Spain: mandatory confinement, measures starting to become less restrictive at T3 Portugal: general duty of home confinement	Parent-report Psychological well-being via Impact Scale of COVID-19 and Home Confinement on Children and Adolescents (anxiety, sleep, mood, behavioural disturbances)	Italy (harder restrictions): Increase in psychopathology (anxiety, negative mood, sleep problems, behavioural and cognitive disturbances) from T1 to T2 Decrease in anxiety and mood symptoms and increase in eating disturbances from T2 to T3 Increase in all symptoms from T1 to T3 Lower symptoms than Spain and Portugal at T1, higher symptoms than Spain and Portugal at T3 Spain (harder restrictions): Increase of anxiety from T1 to T2, then decrease in anxiety from T2 to T3 No changes in other symptoms Portugal (more liberal restrictions): No changes in any symptom category over all waves Lower symptoms than Italy and Spain at T3
37	Paschke et al. [96]	04/2020 (lockdown)	Germany	*M* = 13.6 years 10–17 years	731 adolescent-parent-dyads	T1: 09/2019 T2: 04/2020	Adolescents: Financial worries Procrastination School attendance Emotion regulation problems Parents: Confidence in parenting Stress and emotion regulation problems	Self-report Psychological stress via Perceived Stress Scale (PSS-4)	Increase in psychological stress in parents (29.7%) and adolescents (34.5%) from T1 to T2 Risk factors for stress: female gender, financial worries, parental stress, procrastination, limited access to emotion regulation strategies, mainly staying at home during confinement Protective factor against stress: high emotional awareness
38	Pelham et al. [53]	05–08/2020 (stay-at-home orders)	United States of America	*M* = 12.4 years 10–15 years	7842 adolescents and their parents	T1: 2018–01/2020 T2: 05/2020 T3: 06/2020 T4: 08/2020	Parents’ substance use Pre-existing internalising or externalising problems Worry, stress, material hardship due to pandemic	Self-report Past-30-day substance use Anxiety and depressive symptoms via Patient-Reported Outcomes Measurement Information System (PROMIS) Stress during past month via Perceived Stress Scale (PSS)	Decrease in use of alcohol from T1 to T2–4 Increase in use of nicotine and misuse of prescription drugs from T1 to T2–4 No changes in substance use from T2 to T4 8.0% of youth reported use of any substance, typically only one substance and primarily episodic use Risk factors for substance use: Pre-pandemic externalising symptoms Stress, depression, and anxiety during pandemic Pandemic-related uncertainty, material hardship, parents’ use of alcohol or drugs Associations between risk factors and substance abuse stronger in older adolescents No effects of sex and race/ethnicity
39	Penner et al. [97]	04–06/2020 (stay-at-home orders)	United States of America	*M* = 11.99 years 10–14 years	322 adolescents (mainly Hispanic/ Latinx)	T1: 01/2020 T2-4: 04–06/2020	Effects of the COVID-19 pandemic at home (e.g., financial, physical contact, access to food, stress, family functioning)	Self-report Internalising problems, attention problems, externalising problems via Brief Problem Monitor (BPM)	Statistically and clinically significant decrease in externalising symptoms from T1 to T2 only in adolescents with elevated mental health problems at T1 Statistically but not clinically significant decrease in internalising and total problems from T1 to T2 in whole sample No change in in attention problems from T1 to T2 in whole sample Protective factor against mental health symptoms: better family functioning
40	Qin et al. [98]	04/2020 (lockdown)	China	13–16 years	254 adolescents (high socio-economic status)	T1: 10/2019 T2: 04/2020		Self-report Anxiety (learning anxiety, loneliness anxiety, self-blaming, sensitivity, personal anxiety, phobia, somatic anxiety, impulsive tendencies) via Mental Health Test (MHT)	Increase in number of adolescents with poor mental health from T1 to T2 (12.3% to 24.2%) Increase in learning anxiety, sensitivity tendency, somatic anxiety, phobia tendency No change in personal anxiety, loneliness anxiety, self-blaming tendency, and impulsive tendency Risk factors for poor mental health: no siblings, living in stem family, risk of contracting COVID-19 from family members Protective factor against poor mental health: exercising for minimum 1 h/day
41	Ravens-Sieberer et al. [54]	5–06/2020 (loosening of restrictions after lockdown) and 12/2020–01/2021 (lockdown)	Germany	*M*_T1–T2_ = 12.67 years 7–17 years	T0: 1,556 families T1–2: 1923 families	T0: 2017 (data from different sample) T1: 05–06/2020 T2: 12/2020–01/2021	Parental mental health Family resources/climate Social support	Parent-report for < 11-year-olds, self-report for 11–17-year-olds Health-related quality of life via KIDSCREEN-10 Emotional symptoms, conduct problems, peer problems and hyperactivity via Strengths and Difficulties Questionnaire (SDQ) Anxiety symptoms via Screen for Child Anxiety Related Emotional Disorders (SCARED) Depressive symptoms via Center for Epidemiological Studies Depression Scale (CES-DC) and Patient Health Questionnaire (PHQ-2) Psychosomatic complaints via Health Behavior in School-Aged Children Symptom Checklist 2017 symptom checklist (HBSC-SCL)	Decrease in mental health (increase in anxiety, depression, psychosomatic problems) and health-related quality of life from T0 to T1 and T2 (17.6% suffering from mental health problems at T0 vs. 30.9% at T2), i.e., increase in symptoms from before to during the pandemic Decrease in health-related quality of life from T1 to T2, i.e., over the course of the pandemic No change in global symptoms and conduct problems from T1 to T2 Increase in emotional problems, peer-related mental health problems, anxiety, depressive and psychosomatic symptoms from T1 to T2 Decrease in hyperactivity from T1 to T2 Risk factors for mental health problems: social disadvantage, mentally burdened parents Protective factors against mental health problems: female gender, older age, positive family climate, social support
42	Raw et al. [61]	03–07/2020 (lockdown to loosening of restrictions)	United Kingdom	*M*_T1–T4_ = 9.12–9.25 years 4–16 years	2988 caregivers	T1: 03–04/2020 T2: 04–05/2020 T3: 06/2020 T4: 07/2020	Psychological distress of caregiver Special educational needs, neurodevelopmental disorder Family support	Caregiver-report Emotional symptoms, conduct problems, peer problems, hyperactivity and prosocial behaviour via Strengths and Difficulties Questionnaire (SDQ)	Increase in levels of hyperactivity and conduct problems from T1 to T4 No change in emotional symptoms from T1 to T3, decrease between T3 and T4 Risk factors for elevated symptoms: caregiver psychological distress, special educational needs, neurodevelopmental disorder, younger age
43	Rogers et al. [99]	04/2020 (stay-at-home orders)	United States of America	*M* = 15.24 years 14–17 years	407 adolescents	T1: 10/2019 T2: 04/2020	Loneliness	Self-report Mood via Positive and Negative Affect Schedule (PANAS) Depressive symptoms via Children’s Depression Inventory Short Version (CDI:S) Anxiety symptoms via Generalized Anxiety Disorder Scale (GAD-7)	Increase in negative affect and decrease in positive affect from T1 to T2 Increase in depression, anxiety, and loneliness from T1 to T2 (low mean levels of mental health problems)
44	Romm et al. [100]	03–08/2020 (lockdown to loosening of restrictions)	United States of America	*M* = 15.09 years 14–16 years	208 adolescents (T3 for 123 before 13/03/2022, for 85 after 12/04/2022)	T1-T2: 03/2019–03/2020 T3: 03–08/2020 Until 13/03: pre-COVID-19 group After 12/04: COVID-19 group	Friendship and isolation Emotion regulation strategies: Reappraisal and suppression of positive affect Savouring and dampening of positive affect Eudemonic and hedonic well-being motives	Self-report Depressive symptoms via Children’s Depression Inventory (CDI-2) Positive and negative affect via Positive and Negative Affect Schedule-Short Form (PANAS-SF) Life satisfaction via Students’ Life Satisfaction Scale (SLSS)	Increase in depressive symptoms, negative affect, and isolation from T1 and T2 to T3 only for adolescents in the COVID-19 group Decrease in positive affect and friendship from T1 and T2 to T3 only for adolescents in the COVID-19 group No difference in life satisfaction between COVID-19 group and non-exposed group Risk factors for increased symptoms: dampening positive emotions Protective factors against increased symptoms: eudemonic and hedonic motives
45	Rosen et al. [62]	04–05/2020 (stay-at-home orders, school closures) and 11/2020–01/2021	United States of America	*M* = 12.65 years 7–10; 13–15 years	224 children and adolescents and their caregivers	T1: 01/2016–09/2017 (younger children) and 06/2017–10/2018 (adolescents) T2: 03–11/2018 (younger children only) T3: 04–05/2020 T4: 11/2020–01/2021	Financial, social, school, and physical pandemic-related stressors Potential protective factors, e.g., physical activity, time spent in nature, screen time, family routines	Parent-report Emotional and behavioural problems via Child Behavior Checklist (CBCL) and via Strengths and Difficulties Questionnaire (SDQ) self-report (adolescents) Emotional and behavioural problems via Youth Self Report (YSR) and via Strengths and Difficulties Questionnaire (SDQ)	Increase in internalising and externalising symptoms from T1 and T2 to T3 and T4 Risk factors for increased symptoms: exposure to pandemic-related stressors, pre-pandemic symptoms Protective factors against increased psychopathology and effect of pandemic-related stress factors: structured routine, less passive screen time, lower exposure to news media about the pandemic, more time in nature, adequate sleep
46	Shi et al. [101]	06–07/2020 (re-opening of schools)	China	*M* = 11.74 years 7–17 years	7958 adolescents	T1: 12/2019–01/2020 T2: 06–07/2020	Emotional competence COVID-19 exposure	Self-report Depression via Center for Epidemiological Studies Depression Scale for Children (CES-DC) Anxiety via Screen for Child Anxiety Related Emotional Disorders (SCARED)	Decrease of depression and anxiety from T1 to T2 (38.67% to 36.74%; 13.02% to 12.77%) Emotional competence at T2 as a mediator of effect of COVID-19 exposure on anxiety and depression at T2, i.e., emotional competence had a negative effect on anxiety and depression at T2
47	Shoshani and Kor [102]	05/2020 (re-opening of schools after lockdown)	Israel	*M* = 13.97 years 11.1–17 years	1537 children and adolescents	T1: 09/2019 T2: 05/2020	Daily screen time Perceived social support Daily routines Gratitude	Self-report Anxiety, somatization, panic, and depression via Brief Symptom Inventory-18 (BSI-18) Affect via Positive and Negative Affect Schedule for Children (PANAS-C) Life satisfaction via Brief Multidimensional Students’ Life Satisfaction Scale (BMSLSS)	Increase in anxiety, depression and panic symptoms, video gaming, Internet and TV screen time use from T1 to T2 No change in negative affect from T1 to T2 Decrease in positive emotions, life satisfaction, social media use and peer support from T1 to T2 Risk factor for mental health problems: higher mental health symptoms at T1 Protective factors against mental health problems: consistent daily routines, perceived social support
48	Shum et al. [63]	03/2020–03/2021	United Kingdom	*M*_T1_ = 13.37 years 4–17 years	8752 caretakers, 1284 adolescents	T1: 03/2020 T2–11: monthly reports T12: 03/2021	Household income Neurodevelopmental disorder, special educational needs	Caregiver-report Emotional symptoms, conduct problems and hyperactivity via Strengths and Difficulties Questionnaire (SDQ) Self-report (adolescents) Emotional symptoms, conduct problems and hyperactivity via Strengths and Difficulties Questionnaire (SDQ) Global distress, depressive and anxiety symptoms via Kessler 6 scale (K-6)	Sharp decrease in behavioural, emotional, and attentional difficulties since 02/2021 (ease of restrictions) No reductions of symptoms in children with neurodevelopmental disorders or special educational needs and children from low-income households Highest level of behavioural, emotional, and attentional difficulties in 06/2020 and 02/2021 (highest restrictions) Possible/probable cases of clinically relevant psychopathology in 06/2020 and 02/2021: 20.4–21.7% behavioural, 22.3–24.2% emotional, 28.6–29.4% attentional Younger children showed greater changes in levels of psychological difficulties than older children/adolescents No gender differences in patterns of symptomology over time, higher levels of attentional problems reported for boys and higher levels of emotional problems reported for girls
49	Skripkauskaite et al. [64] (same project as Shum et al. [63])	03/2020–06/2021	United Kingdom	*M*_T1_ = 13.37 years 4–18 years	9161 caretakers	T1: 03/2020 T2–14: monthly reports T15: 06/2020	Household income Neurodevelopmental disorder, special educational needs	Caregiver-report Emotional symptoms, conduct problems and hyperactivity via Strengths and Difficulties Questionnaire (SDQ)	See: Shum et al. 2021 No change in behavioural, emotional, and attentional difficulties between 04 and 06/2021 Possible/probable cases of clinically relevant psychopathology in 06/2021: 19.5% behavioural, 21.8% emotional, 24.1% attentional
50	Specht et al. [103]	04/2020 (lockdown)	Denmark	*M* = 5.0 years 3.5–6.8 years	40 parents	T1: 01–02/2020 T2: 04/2020	Leisure time activities	Caregiver-report Externalising (conduct problems and hyperactivity), internalising symptoms (emotional symptoms, peer problems), and prosocial behaviour via Strengths and Difficulties Questionnaire (SDQ)	Increase in total difficulty score and externalising symptoms (i.e., hyperactivity) from T1 to T2 No change in internalising symptoms (i.e., emotional symptoms and peer problems) and conduct problems from T1 to T2 Decrease of prosocial behaviour from T1 to T2 Risk factor for mental health problems at T2: attending leisure time activities prior to lockdown
51	Spencer et al. [104]	08/2020–01/2021	United States of America	*M* = 8.5 years 5–11 years at T1	168 caregivers (mostly racial and ethnic minorities)	T1: 09/2019–03/2020 T2: 08/2020–01/2021	Social risks (e.g., food and housing insecurity, unemployment, medication affordability) Caregiver mental health COVID-19-related stressors (e.g., remote school assignments, exposure to COVID-19-related media, screen time use)	Caregiver-report Attention, internalising and externalising symptoms via Paediatric Symptom Checklist (PSC-17)	Increase in total score from T1 to T2 (increase in overall risk of mental health problems from 8 to 18%) Increase in attention, internalizing and externalising symptoms from T1 to T2 (increase in clinically relevant internalising symptoms from 5 to 18%) Factors associated with increased mental health symptoms: number of social risks before pandemic, less school assignment completion, increased screen time, caregiver depression
52	Teng et al. [105]	04–05/2020 (stay-at-home orders)	China	Grade 4 and 7 ~ 9–10 years and 12–13 years	1778 children and adolescents	T1: 10–11/2019 T2: 04–05/2020	Perceived COVID-19 impacts Videogame use	Self-report Internet Gaming Disorder via Internet Gaming Disorder Scale-Short Form (IGDS9-SF) Depressive symptoms via Center for Epidemiologic Studies Depression Scale (CES-D) Anxiety symptoms via State-Trait Anxiety Inventory (STAI)	Increase in videogame use and anxiety from T1 to T2 in total sample No change in depressive symptoms from T1 to T2 in total sample Increase in Internet Gaming Disorder from T1 to T2 only in adolescents No change in anxiety and Internet Gaming Disorder from T1 to T2 in children Depressive and anxiety symptoms at T1 predicted Internet Gaming Disorder and videogame use at T2 (mediated by perceived COVID-19 impacts)
53	van der Laan et al. [106]	04/2020 (five weeks after introduction of lockdown)	Netherlands	*M* = 15.53 years 12–16 years	158 adolescents	T1: 03/2019 T2: 04/2020	Concerns about COVID-19	Self-report Life satisfaction via Cantril Ladder Internalising symptoms via Revised Child Anxiety and Depression Scale (RCADS) Psychosomatic health via Health Behavior in School-Aged Children Symptom Checklist 2017 (HBSC)	Decrease in life satisfaction from T1 to T2 Increase in psychosomatic health from T1 to T2 No change in internalising symptoms from 1 to T2 Gender as a moderating factor: Higher life satisfaction and psychosomatic health, and lower internalising symptoms in boys than in girls at T1 and T2 Decrease in life satisfaction from T1 to T2 only in boys, not in girls Risk factor for lower life satisfaction: concerns about COVID-19
54	Vira and Skoog [107]	11/2020–02/2021 (schools up to grade 6 remained open during the pandemic)	Sweden	*M*_T1_ = 10 years *M*_T2_ = 11 years 9–12 years	849 children	T1: 10/2019–01/2020 T2: 11/2020–02/2021	Relationships to others (social support through parents and friends) School adjustment (social support through teachers, school, and class well-being)	Self-report Emotional problems via Strengths and Difficulties Questionnaire (SDQ) Sense of hope via Children’s Hope Scale (CHS) Self-efficacy via Children’s Self-Efficacy Scale (CSES) Self-esteem via Self-Esteem Scale (S/SE)	Negligible decrease in psychological adjustment (i.e., sense of hope, self-efficacy, self-esteem, and emotional problems) from T1 to T2 No changes in emotional problems from T1 to T2 Decrease in school adjustment from T1 to T2 (slightly less social support by teachers, slightly less well-being at school) No changes in relationship to others from T1 to T2
55	Walters et al. [108]	11/2020 (most schools offering hybrid instruction (remote and in-person))	United States of America	*M* = 12.38 years	174 adolescents	T1-T3: 2016–2018 T4: 11/2019 T5: 11/2020	Neutralizing beliefs Bullying victimization and perpetration Parental support and knowledge	Self-report Depression via 5-item Center for Epidemiological Studies Depression Scale (CES-D) Cognitive impulsivity via Weinberger Adjustment Inventory Impulse Control Scale (WAI-IC) Delinquency via Self-Reported Offending Scale (SRO)	No changes in depression, cognitive impulsivity, delinquency, bullying victimization and perpetration, and parental knowledge from T4 to T5 Modest increase in neutralization beliefs, and small decrease in parental support from T4 to T5 Smaller changes from T4 to T5 than expected based on data from T1-T4 (i.e., decrease in parental support and knowledge, increase of delinquency and bullying victimisation)
56	Wright et al. [109]	06–08/2020 (re-opening after lockdown, social distancing measures)	United Kingdom	*M* = 11.97 years 10–12 years	202 children and their mothers	T1: 12/2019–02/2020 T2: 06–08/2020	Children’s long-term vulnerability: Prior adjustment Maternal prenatal depression Neighbourhood deprivation COVID-19-related experiences: Parent in frontline job Financial difficulties during pandemic Stressful events during the pandemic	Parent- and self-report Depression via Short Mood and Feelings Questionnaire (SMFQ) PTSD via Child Trauma Symptom Scale parent-report Anxiety via Short Spence Anxiety Scale (SCAS-S) Behavioural problems via Child Behavior Checklist (CBCL)	44% increase in symptoms of depression from T1 to T2 26% increase in post-traumatic stress disorder from T1 to T2 76% increase in disruptive behaviour problems (particularly in children without previous externalising symptoms) from T1 to T2 No change in anxiety from T1 to T2 Changes only in group of less deprived families Risk factors for depression at T2 and greater absolute increases from T1 to T2: female gender, higher internalising symptoms earlier in childhood No influence of COVID-19 related experiences
57	Wu et al. [110]	05/2020 (post-lockdown)	China	*M* = 12.7 years	1627 adolescents	T1: 10/2019 T2: 05/2020		Self-report Psychotic-like experiences, anxiety, depression via Mental Health Inventory of Middle School Students (MMHI-60)	Increase in psychotic experiences, anxiety, and depression from T1 to T2 Positive correlation between changes of psychotic experiences and changes in anxiety and depression Greatest exacerbation in anxiety and depression symptoms in group with new-onset psychotic experiences at T2 (16.7%)
58	Wunsch et al. [111]	04/2020 (lockdown)	Germany	*M* = 10.36 years 4–17 years	1711 children and adolescents and their parents	T1: 08/2018–03/2020 T2: 04/2020	Physical activity Screen time	Parent-report for < 11 years, self-report for 11–17 years Health-related quality of life via KIDSCREEN-10	Decrease in health-related quality of life from T1 to T2, relatively low scores compared to European norms Increase of physical activity and screen time from T1 to T2 Physical activity and screen time at T1 not predictive of health-related quality of life at T2 Screen time and health-related quality of life at T1 as predictors of physical activity at T2

This table summarises the main characteristics and findings of the longitudinal studies (*n* = 58). The studies are sorted alphabetically based on the first author’s last name. Two studies listed here combined longitudinal (within-subjects) and repeated cross-sectional (between-subjects) designs [53, 54]. The studies of Shum et al. [63] and Skripkauskaite et al. [64] belong to the same longitudinal project and thus report partially on the same study sample. We summarised additional variables (“COVID-19-related stressors/Other variables”) and the table does not provide a complete record of all studied variables. As most studies collected demographic information like age, gender and socio-economic background, these variables are not specifically listed as “other variables” in the table. If not specified otherwise, the depicted results include only findings concerning children and adolescents

**Table 2:** Overview of repeated cross-sectional studies (different samples)

No	Authors	Assessment during pandemic	Country	*M*_Age_/Age range	*N*	Waves of assessment	COVID-19-related stressors/Other variables	Assessment of children’s and adolescents’ mental health symptoms	Main findings
1	Burdzovic Andreas and Brunborg [112]	10–12/2020 (implementation of stricter national policies)	Norway	Grade 11 ~ 16 years	Adolescents T1: 1621 T2: 915	T1: 10–12/2018 and 10–12/2019 T2: 10–12/2020	Physical health Participation in Organized sports number of friends	Self-report Depression symptoms via Patient Health Questionnaire-9 (PHQ-9) Pandemic anxiety via Pandemic Anxiety Scale (CPAS-11; only T2)	No differences in depression and physical health between T1 and T2 17.3% reported high pandemic anxiety at T2 Adolescents with high pandemic anxiety showed higher depression symptoms, poorer physical health, and less participation in organized sports
2	Dabravolskaj et al. [113]	11–12/2020 (post-lockdown, reopening of schools)	Canada	Grade 4–6 9–12 years	Children T1: 476 T2: 467 (low socio-economic status)	T1: spring 2018 T2: 11–12/2020		Self-report Mental health and well-being via 12 questions about worries, unhappiness, sadness, relationships, schoolwork, self-confidence, feeling of belonging, etc	No significant differences in well-being and mental health from T1 to T2
3	Dollberg et al. [114]	03–04/2020 (lockdown)	Israel	*M* = 4.17 years 3–6 years	Mothers T1: 87 T2: 53	T1: pre-pandemic T2: 03–04/2020	Mothers’ anxiety Maternal mentalization/mind-mindedness	Parent-report Children’s internalising and externalising behaviour via Child Behavior Checklist 1.5–5 (CBCL 1.5–5)	Higher children’s externalising and internalising behaviour, and higher maternal anxiety at T2 than at T1 Mothers’ anxiety as mediator of the effect of pandemic on children’s externalising and internalising symptoms Mothers’ mind-mindedness as moderator of the association between the pandemic and children’s externalising behaviours
4	James et al. [115]	04–06/2020 (school closures)	United Kingdom	*M*_T1_ = 10.3 years *M*_T2_ = 10.27 years *M*_T3_ = 9.99 years 7–11 years	Children T1: 475 T2: 1150 T3: 1333	T1: 04–06/2018 T2: 04–06/2019 T3: 04–06/2020	Physical activity Screen time Diet and dental health Competency and autonomy Socioeconomic deprivation	Self-report Well-being via study-intern questionnaire Emotional and behavioural difficulties via study-intern questionnaire	Higher physical activity levels, sleep time, happiness, and general well-being at T3 than at T1 and T2 Lower levels of emotional and behavioural symptoms at T3 than at T1 and T2 Less school competence and physical health behaviours (healthy diet, physical activity) in socioeconomically deprived children than in children with a higher socio-economic status at T3
5	D. Kim and Lee [116]	11–12/2020 (partial school closures)	South Korea	*M*_T1_ = 13.36 years *M*_T2_ = 13.62 years ~ 10–15 years	Adolescents T1: 3040 T2: 2906	T1: 11–12/2018 T2: 11–12/2020	Time spent playing games on the Internet	Self-report Internet Gaming Disorder via Maladaptive Gaming Usage Scale (MGUS)	Higher addictive internet gaming at T2 than at T1 (1.2% to 4.9%, little clinical significance) More time spent on gaming at T2 than at T1, especially in subjects with higher scores of addictive gameplay Males more likely to display higher addictive Internet gaming usage
6	S. Y. Kim et al. [117]	08–11/2020 (partial school closures)	South Korea	*M*_T1_ = 15.0 years *M*_T2_ = 15.1 years 12–18 years	Adolescents T1: 48,443 T2: 44,216	T1: 06–07/2019 T2: 08–11/2020	Physical activity Sedentary time Sleep time Socio-economic status Health status Scholastic performance	Self-report Subjective stress level via items created for the survey Sadness or despair via items created for the survey Suicidal thoughts/planning/ attempt via items created for the survey	Lower degree of severe subjective stress, sadness and despair, and fewer suicidal behaviours at T2 than at T1 Lower physical activity and sleep duration, and higher sedentary time at T2 than at T1 No difference in scholastic performance and subjective health between T1 and T2 No effect of scholastic performance, socio-economic status, and gender on differences in mental health between T1 and T2
7	Koenig et al. [118]	03–08/2020 (lockdown, school closures)	Germany	*M* = 14.93 years ≥ 12 years	Adolescents T1: 324 T2: 324 (matched convenience sample)	T1: 11/2018–03/2020 T2: 03–08/2020	Psychosocial risk factors Socio-economic status Family risk factors	Self-report Emotional symptoms, conduct problems, peer problems, hyperactivity and prosocial behaviour via Strengths and Difficulties Questionnaire (SDQ) Depression via Patient Health Questionnaire modified for Adolescents (PHQ-A) Eating disorder symptoms via Weight Concerns Scale (WCS) and Eating Disorder Examination Questionnaire (EDE-Q) Health-related quality of life via KIDSCREEN-10 Suicidal thoughts and behaviours via Paykel Suicide Scale (PSS)	No significant difference in emotional or behavioural problems, depression, suicidal thoughts/attempts, eating disorder symptoms, or general health-related quality of life between T1 and T2 Lower rated of suicide planning (6.14% to 2.16%) and conduct problems at T2 than at T1 Family risk factors did not moderate effects of pandemic on mental health Decrease of influence of socio-economic status on mental health problems from T1 to T2
8	Lane et al. [119]	10–12/2020 (lockdown)	Canada	*M*_T1_ = 12.66 years *M*_T2_ = 12.59 years Grade 7–8 ~ 12–14 years	Adolescents T1: 1610 T2: 1380	T1: 10–12/2019 T2: 10–12/2020		Self-report Depression via Children’s Depression Inventory (CDI) Problematic internet use via Compulsive Internet Use Scale (CIUS) Symptoms of various anxiety disorders (e.g., GAD, PTSD, social anxiety disorder etc.) via Screen for Child Anxiety Related Emotional Disorders Revised (SCARED) Test anxiety via Test Anxiety Inventory – Short Form (TAI) Fear of being judged via Fear of Negative Evaluation Scale (FNE) Perfectionism via Child-Adolescent Perfectionism Scale Intolerance to uncertainty, cognitive avoidance and negative attitude when faced with problems via Cognitions Reliées à l’Anxiété Généralisée pour Enfants Questionnaire (CAG) Impact of anxiety via Child/Adolescent Anxiety Impact Scale (CAIS)	More symptoms of generalized anxiety, test anxiety, fear of judgement and perfectionism reported at T2 than at T1 No difference in all other variables (e.g., PTSD, social anxiety disorder, internet use, depression) between T1 and T2 14.4% reported that lockdown had a positive effect on their well-being and mental health, 23.5% reported negative effect, 62.1% reported no effect More students in grade 8 than in grade 7 and more girls than boys reported negative effect of lockdown on mental health
9	Luijten et al. [120]	04–05/2020 (lockdown)	Netherlands	*M*_T1_ = 13.1 years *M*_T2_ = 13.4 years 8–18 years	Children and adolescents T1: 2,401 T2: 844 and their parents	T1: 12/2017–07/2018 T2: 04–05/2020	Change in parental working situation COVID-19 infection in a friend or family member Attendance of childcare/school during lockdown Family atmosphere during lockdown and before Impact of lockdown on daily life	Self-report Global health, peer relationships, anxiety, depressive symptoms, anger, sleep-related impairment via Patient-Reported Outcomes Measurement Information System (PROMIS)	Worse scores in all domains at T2 than at T1, i.e., more severe anxiety (8.6–16.7%), depression and sleep-related impairment (7.1–8.2%) Less reports of poor global health (T1: 4.6% to T2: 1.7%) Worse atmosphere in the family during lockdown than before 90% indicated that lockdown had a negative impact on their life (missing friends and family, participation in sports, freedom, school and joyful activities, and boredom) Risk factors for mental health problems: single-parent family, more than two children in the family, negative change in working situation of parents due to COVID-19, infected friend or relative
10	Saito et al. [65]	05/2020 (school closure) and 10/2020 (school reopening)	Japan	*M* = 11.4 years ≥ 9 years	Children and adolescents T1: 78 T2: 113	T1: 05/2020 T2: 10/2020	Learning environment Time spent with family members Contact with friends Changes in daily routines Physical condition Home activities	Self-report Well-being via WHO Five Well-Being Index (WHO-5-J): learning environment, contact with friends and family, home activities, daily routine, and physical condition	No change number of children with mental health problems from T1 to T2 (18–20%) More disruptions of sleep rhythms, eating habits and physical activity at T1 than at T2 More time spent with family and sleeping, and more rest at T1 than at T2 Less activity and interest at T1 than at T2
11	Thorisdottir et al. [121]	09–11/2020 (strict restrictions, third wave of pandemic)	Iceland	13–18 years	Adolescents T1: 21,404 T2: 20,822 T3: 17,475	T1: 02/2016 and 10/2016 T2: 02/2016 and 10/2018 T3: 09–11/2020		Self-report Depressive symptoms via Symptom Checklist-90 (SCL-90) Mental well-being via Short Warwick Edinburgh Mental Well-being Scale (WEMWBS) Frequency of cigarette smoking, e-cigarette use and alcoholic intoxication	Increase depressive symptoms and worsening in mental well-being from T1 and T2 to T3 across all age groups Decrease in cigarette smoking, e-cigarette use and alcohol intoxication among 15–18-year-olds Worse mental health outcomes in girls than in boys, no gender differences in substance use

This table summarises the main characteristics and findings of repeated cross-sectional studies (*n* = 11). The studies are sorted alphabetically based on the first author’s last name. The studies combining longitudinal (within-subjects) and repeated cross-sectional (between-subjects) designs (*n* = 2) are listed in Table 1. We summarised additional variables (“COVID-19-related stressors/other variables”) and the table does not provide a complete record of all studied variables. As most studies collected demographic information like age, gender, and socio-economic background, these variables are not specifically listed as “other variables” in the table. If not specified otherwise, the depicted results include only findings concerning children and adolescents

### Changes in mental health due to the COVID-19 pandemic

Table 3 displays the mental health indicators and symptoms assessed in the studies with a pre-pandemic measurement and how frequently the respective indicators and symptoms were assessed. Table 3 also summarises the studies’ findings concerning the change of the prevalence or intensity of the respective mental health indicators and symptoms from before the pandemic to during the pandemic. The seven studies without a pre-pandemic measure are not included in the table [56, 59–61, 63–65].

**Table 3:** Summary of main results of studies comparing pre-pandemic levels of mental health with measures during the pandemic

Indicator of mental health	Number of studies (*N*)	Increase from pre-pandemic to pandemic	No change from pre-pandemic to pandemic	Decrease from pre-pandemic to pandemic
Quality of life/well-being	11	2 [83, 115]	2 [113, 118]	8 [54, 70, 76, 83, 94, 95, 111, 121]
Life satisfaction	3	0	1 [100]	2 [102, 106]
Resilience	1	0	0	1 [91]
Positive affect	4	0	1 [80]	3 [99, 100, 102]
**Total indicators of good mental health**		**2**	**4**	**14**
Global measures of mental health problems	8	5 [73, 81, 103, 104, 107]	3 [73, 90, 113]	1 [97]
Stress	5	4 [57, 75, 76, 96]	0	1 [117]
Negative affect	4	3 [80, 99, 100]	1 [102]	0
Internalising symptoms	21	8 [62, 78, 82, 84, 88, 89, 104, 114]	9 [66, 67, 71, 72, 90, 103, 106, 107, 118]	5 [81, 88, 92, 97, 115]
Externalising symptoms	13	8 [62, 82, 84, 89, 103, 104, 109, 114]	4 [66, 67, 97, 118]	2 [97, 115]
Depression symptoms	29	19 [54, 55, 68–70, 72, 77, 79, 85–87, 93, 99, 100, 102, 109, 110, 120, 121]	7 [57, 84, 105, 108, 112, 118, 119]	3 [71, 101, 117]
Anxiety symptoms	20	13 [54, 55, 70, 77, 79, 86, 98, 99, 102, 105, 110, 119, 120]	8 [57, 58, 72, 77, 98, 105, 109, 119]	5 [57, 58, 71, 87, 101]
Cognitive symptoms	7	5 [55, 78, 84, 92, 104]	2 [81, 97]	0
Hyperactivity symptoms	8	5 [78, 84, 88, 90, 103]	3 [55, 81, 84]	1 [88]
Disruptive behaviour symptoms	9	4 [55, 81, 84, 88]	6 [55, 78, 84, 90, 103, 108]	2 [88, 118]
Behavioural addiction symptoms (electronic media, Internet)	5	3 [57, 105, 116]	1 [105]	1 [75]
Substance use and addiction symptoms	3	1 [53]	1 [74]	3 [53, 74, 121]
Psychosomatic symptoms	4	3 [54, 76, 92]	0	1 [106]
Post-traumatic stress symptoms	2	1 [109]	1 [119]	0
Suicidal symptoms	2	0	1 [118]	2 [117, 118]
Neurotic symptoms	1	1 [68]	0	0
Dissociative symptoms	1	0	1 [68]	0
Eating disorder symptoms	1	0	1 [118]	0
Psychotic symptoms	1	1 [110]	0	0
**Total indicators of mental health problems**		**84**	**49**	**27**

This table depicts the number of studies investigating a respective indicator of mental health; the number of studies finding a significant increase, significant decrease, and/or no change of a respective indicator from a pre-pandemic assessment to an assessment during the COVID-19 pandemic; and the sum of findings over all indicators of good mental health/mental health problems. Studies with no pre-pandemic measurement are not included in this table (*n* = 7). Studies appear multiple times in the same category if they reported multiple trends, e.g., for different age groups or for different aspect of a symptom group. If a study assessed multiple outcomes, each finding is listed separately.

The results of the 14 studies that investigated changes over the course of the pandemic are mixed. The overall tendency, however, indicates a decrease in mental health symptoms (internalising and externalising symptoms, stress, depression, anxiety, conduct problems, attention problems, problematic smartphone and Internet use) and an increase in well-being when we compared assessments taken during periods of lockdown/stronger restrictions and higher infection rates with those taken during or after the lift of restrictions and easing of the pandemic situation [55–57, 59–61, 63, 64, 66]. However, eight studies did not find a significant change in certain symptoms (i.e., internalising and externalising symptoms, depression, anxiety, attention problems, substance use) and thus do not completely support this trend [53, 55, 57–61, 65]. Two of those studies even reported an increase in certain mental health symptoms (i.e., eating disorder symptoms, hyperactivity, conduct problems) [60, 61]. One study that compared measurements between a period of lifted regulations and a following period of reinforced restrictions reported mainly an increase in symptoms of poor mental health (i.e., anxiety, depression, and psychosomatic symptoms, decrease in health-related quality of life) [54].

Although most studies reported a decrease in at least some psychopathological symptoms after an ease of the pandemic situation, it is less clear if children and adolescents return to a pre-pandemic level of functioning. Of the seven studies with both a pre-pandemic measurement and multiple during-pandemic measurements, five reported an increase in some symptoms from the pre-pandemic measure to assessment during loosened restrictions within the pandemic [53–55, 57, 62]. Three studies reported a decrease in symptoms from pre-pandemic levels to during less-restricted periods of the pandemic [53, 57, 58]. Three studies did not find a difference between the two time points [55, 57, 66]. Note that some of the seven studies assessed multiple outcomes and reported multiple directions of change (e.g., decrease in depression, anxiety, sluggish cognitive tempo and oppositional/defiant symptoms and no change in hyperactivity and impulsivity symptoms from spring 2020 to summer 2020 [55]).

### Factors that influence COVID-19-related changes in mental health

Table 4 summarises factors reported to be associated with mental health outcomes during the COVID-19 pandemic. For simplicity, we classified the factors as “risk factors” if they were associated with more negative mental health effects and as “protective factors” if they were associated with less severe negative mental health outcomes.

**Table 4:** Risk and protective factors of the COVID-19 pandemic’s mental health effects

Factor categories	Risk factors	Protective factors
Sociodemographic factors	Female gender (depression, anxiety, internalising symptoms, well-being, life satisfaction, stress, psychosomatic symptoms) [63, 85–88, 94, 96, 106, 109, 119, 121] Male gender (general mental health problems, quality of life, attention problems, addictive behaviour) [54, 63, 73, 116] Younger age (behavioural and emotional problems, psychosomatic symptoms, quality of life) [54, 61, 63] Older age (depression, anxiety, behavioural addiction) [53, 58, 84, 92, 105, 119] Being an only child [88, 98] Low family income, financial worries [55, 82, 88, 96] Social disadvantage [54, 104] Living in an apartment, without access to green space [94]	Female gender (general mental health problems, quality of life, attention problems, addictive behaviour) [54, 63, 73, 116] Male gender (depression, anxiety, internalising symptoms, well-being, life satisfaction, stress, psychosomatic symptoms) [63, 85–88, 94, 96, 106, 109, 119, 121] Younger age (depression, anxiety, behavioural addiction) [53, 58, 84, 92, 105, 119] Older age (internalising and externalising problems, quality of life) [54, 61, 63] Living in a house, access to green space [94]
Intra-individual factors	Pre-existing mental health problems [88, 102, 109] Poor physical health [75] Neurodevelopmental disorders, special educational needs [61, 63] Negative coping strategies (emotion-focussed engaged and disengaged coping) [67, 89] Dysfunctional/missing emotion regulation abilities (e.g., dampening positive emotions) [55, 80, 96, 100] Negative cognitive styles [85] Emotional instability [68] High extraversion [69]	Good physical health [75] Positive coping mechanisms (e.g., talking to friends, prioritising sleep, problem-focussed engaged coping) [77] Positive emotion regulation abilities, high emotional awareness/competence [55, 80, 96, 101] Resilience [85, 91] Self-efficacy [89] Eudemonic and hedonic motives [100]
Parental/family factors	Single parent [88, 98, 120] Parental stress and strain [56, 59, 96] Parental mental health problems, psychological distress [54, 61] Depression [72, 104] Anxiety [114] Use of alcohol and drugs [53] Parental negative coping strategies [67] Maternal burden of care [72] Parental over-reactivity [67] Parental expressive suppression of emotions [59] Dysfunctional parenting styles [81] Family stress and instability [92]	Family functioning [97] Positive family climate [54] Maternal high mind-mindedness [114]
Social factors	Deterioration of relationships to others [81] Missing friends [92]	Friend support/contact to friends [71, 72, 87, 91] Social support [54, 91, 102]
COVID-19-related factors	Infection risk, infected friend or relative [98, 120] Perceived lifestyle impact of the pandemic [79] Negative change in parental working situation [120] Perceived stress [67, 85, 87] Isolation and loneliness [68, 69] Worry, concerns about health [68, 81, 87, 92] Concerns/fears about the pandemic [106, 112] Frustration, boredom [81] Negative home-schooling experience [92] Increased restrictions [59, 60] Feelings of uncertainty [53] Material hardship [53] High exposure to news [62]	
Behavioural factors	Mainly staying at home during confinement [96] Increased, passive screen time [62, 104] Taking part in many leisure time activities prior to the pandemic [103] Less school assignment completion [104]	Physical health behaviours: Exercising for minimum 1 h/day [98] Adequate sleep [62] Structured routines [62, 102] Time spent in nature [62]

Table 5 portrays the change of mental health indicators and symptoms from before to during the pandemic and how frequently these symptoms were assessed in three separate age groups (< 6 years, six–12 years, > 12 years).

**Table 5:** Summary of the main results of studies that compare pre-pandemic levels of mental health with levels measured during the pandemic sorted by age group

Indicator of mental health	Direction of change	Younger children (~ < 6 years)	School-aged children (~ 6–12 years)	Adolescents (~ > 12 years)
Quality of life/well-being	+	0	1 [115]	1 [83]
	0	0	1 [113]	1 [118]
	–	1 [111]	3 [54, 94, 111]	8 [54, 70, 76, 83, 94, 95, 121, 111]
Life satisfaction	+	0	0	0
	0	0	0	1 [100]
	–	0	0	2 [102, 106]
Resilience	+	0	0	0
	0	0	0	0
	–	0	0	1 [91]
Positive affect	+	0	0	0
	0	0	1 [80]	1 [80]
	–	0	0	3 [99, 100, 102]
**Total indicators of good mental health**	+	0	1	1
	0	0	2	3
	–	1	3	14
Global measures of mental health problems	+	2 [73, 103]	3 [73, 104, 107]	2 [81, 98]
	0	2 [73, 90]	2 [73, 113]	0
	–	0	1 [97]	1 [97]
Stress	+	0	3 [57, 75, 96]	3 [57, 76, 96]
	0	0	0	0
	–	0	0	1 [117]
Negative affect	+	0	1 [80]	3 [80, 99, 100]
	0	0	0	1 [102]
	–	0	0	0
Internalising symptoms	+	1 [114]	5 [62, 82, 84, 88, 104]	4 [62, 78, 88, 89]
	0	3 [84, 90, 103]	5 [66, 67, 71, 72, 107]	3 [66, 106, 118]
	–	0	4 [88, 92, 97, 115]	3 [81, 88, 97]
Externalising symptoms	+	2 [103, 114]	5 [62, 82, 84, 104, 109]	2 [62, 89]
	0	1 [84]	3 [66, 67, 97]	3 [66, 97, 118]
	–	0	2 [97, 115]	1 [97]
Depression symptoms	+	0	4 [54, 72, 109, 120]	17 [54, 55, 68–70, 77, 79, 85–87, 93, 99, 100, 102, 110, 120, 121]
	0	1 [84]	3 [57, 84, 105]	7 [57, 77, 105, 108, 112, 118, 119]
	–	0	2 [71, 101]	2 [101, 116]
Anxiety symptoms	+	0	2 [54, 120]	13 [54, 55, 70, 77, 79, 86, 98, 99, 102, 105, 110, 119, 120]
	0	0	5 [57, 58, 72, 105, 109]	4 [57, 77, 98, 119]
	–	0	4 [57, 58, 71, 101]	3 [57, 87, 101]
Cognitive symptoms	+	0	3 [84, 92, 104]	2 [55, 78]
	0	0	1 [97]	2 [81, 97]
	–	0	0	0
Hyperactivity symptoms	+	2 [90, 103]	2 [84, 88]	2 [78, 88]
	0	1 [84]	0	2 [55, 81]
	–	0	1 [88]	1 [88]
Disruptive behaviour symptoms	+	0	3 [84, 88, 109]	3 [55, 81, 88]
	0	3 [84, 90, 103]	2 [84]	3 [55, 78, 108]
	–	0	1 [88]	2 [88, 118]
Behavioural addiction symptoms (electronic media, Internet)	+	0	2 [57, 116]	3 [57, 105, 116]
	0	0	1 [105]	0
	–	0	1 [75]	0
Substance use and addiction symptoms	+	0	1 [53]	1 [53]
	0	0	0	1 [74]
	–	0	1 [53]	3 [53, 74, 121]
Psychosomatic symptoms	+	0	2 [54, 92]	2 [54, 76]
	0	0	0	0
	–	0	0	1 [106]
Post-traumatic stress symptoms	+	0	1 [109]	0
	0	0	0	1 [119]
	–	0	0	0
Suicidal symptoms	+	0	0	0
	0	0	0	1 [118]
	–	0	0	2 [117, 118]
Neurotic symptoms	+	0	0	1 [68]
	0	0	0	0
	–	0	0	0
Dissociative symptoms	+	0	0	0
	0	0	0	1 [68]
	–	0	0	0
Eating disorder symptoms	+	0	0	0
	0	0	0	1 [118]
	–	0	0	0
Psychotic symptoms	+	0	0	1 [110]
	0	0	0	0
	–	0	0	0
**Total indicators of mental health problems**	+	7	37	59
	0	11	22	30
	–	0	17	20

The numbers of studies that found a significant increase (indicated by “ + ”), significant decrease (indicated by “–”), and no change (indicated by “0”) of a respective indicator from a pre-pandemic assessment to an assessment during the COVID-19 pandemic and the sum of findings over all indicators of good mental health/mental health problems are sorted by age of participants. Studies with no pre-pandemic measurement are not included in this table (*n* = 7). Studies that reported multiple trends, e.g., for different age groups or for different aspects of a symptom group appear multiple times in the same category. For studies that assessed multiple outcomes, each finding is listed separately

Table 6 summarises regional differences in mental health outcomes by listing the countries with more than three studies in this review.

**Table 6:** Change of mental health from pre-pandemic to during the pandemic sorted by country

Country	Number of studies (*N*)	Worsening in mental health from pre-pandemic to pandemic	No change in mental health from pre-pandemic to pandemic	Improvement of mental health from pre-pandemic to pandemic
United States of America	14	11 [53, 55, 62, 77, 80, 82, 85, 89, 99, 100, 104]	7 [55, 74, 77, 80, 97, 100, 108]	3 [53, 74, 97]
China	8	7 [57, 75, 91, 93, 98, 105, 110]	3 [57, 98, 105]	3 [57, 75, 101]
United Kingdom	4	3 [72, 88, 109]	2 [72, 109]	2 [88, 115]
Germany	5	5 [54, 69, 96, 111]	1 [118]	1 [118]
Canada	6	5 [70, 73, 79, 87, 119]	3 [73, 113, 119]	1 [87]
Netherlands	4	3 [71, 106, 120]	3 [67, 71, 106]	1 [106]
Spain	4	3 [60, 81, 84]	4 [58, 60, 81, 84]	2 [58, 81]

The number of findings on a respective direction of change of any mental health symptom or indicator from before to during the pandemic are sorted by country. Only countries with at least four studies are included. Three studies from the United Kingdom and one study from Germany without a pre-pandemic measure are not included in this table [56, 61, 63, 64]. Studies appear multiple times if they reported multiple trends, e.g., for different age groups or for different indicators

## Discussion

The COVID-19 pandemic and the protection measures to contain its spread have massively changed the daily lives of billions of children and adolescents worldwide. Here we present a scoping literature review on the longitudinal effects of the COVID-19 pandemic on child and adolescent mental health. There is a lack of reviews and meta-analyses that (a) investigate longitudinal changes in child and adolescent mental health, and (b) include studies conducted after the initial months of the pandemic. We executed the current review according to PRISMA-ScR [46].

Sixty-nine longitudinal and repeated cross-sectional studies published between September 2020 and December 2021 assessing child and adolescent mental health over the first 17 months of the pandemic are included. We have assessed changes in a range of both broad mental health indicators and specific psychopathological symptoms from before to during the pandemic and over its course. Furthermore, we summarised factors that influenced these effects.

### Changes in mental health from before to during the pandemic

Among﻿﻿ all the indicators of mental health problems, such as psychological distress, negative affect, and specific psychopathological symptoms, 84 findings of an increase in a single mental health indicator were reported. However, almost as many findings did not support an increase in mental health problems, with 49 showing no change and 27 showing a decrease in such an indicator. The findings for the change of broad measures of poor mental health (i.e., outcomes not specific to a certain indicator or symptom group, e.g., total scores of mental health questionnaires) are also mixed but more in line with an increase in these constructs. Five findings support this trend compared to four that do not. On the other hand, there is clearer support for an increase in the indicators “stress” and “negative affect”. Each had only one finding not supportive of an increase, compared to four and three findings, respectively, that support an increase.

The findings for indicators of good mental health, such as quality of life, life satisfaction, positive affect, resilience, and well-being, are more homogeneous and support a decrease in mental health from before to during the pandemic (14 supportive findings vs.  six non-supportive findings). The trend for global measures of internalising symptoms is less clear and would rather suggest no change (nine supportive findings) or an increase (eight supportive findings) than a decrease in symptoms (five supportive findings).

Negative effects of the pandemic are clearer if symptoms of individual internalising syndromes are inspected separately. The support for an increase in depressive symptoms is strong (19 supportive findings vs. 10 non-supportive findings). This increase fits the presumed effect based on previous literature and appears logical in the face of enforced isolation, lack of social contacts, pandemic-related worries, and loss of many positive leisure time activities and daily structure. However, there is more variability in data regarding anxiety symptoms with 13 studies finding an increase in symptoms and the same number of studies not reporting an increase in symptoms.

A rise in the general scores of externalising symptoms from before to during the COVID-19 pandemic is supported by most of the reviewed studies assessing these symptoms (eight supporting findings vs. six non-supporting findings). When specific externalising syndromes are regarded separately, different results can be observed. While symptoms of hyperactivity and inattention seem to have increased (hyperactivity: five supportive findings vs. four non-supportive; inattention: five supportive findings vs. two non-supportive), there is greater evidence for no change in the prevalence of disruptive behaviour symptoms (six supportive findings) than for an increase (four supportive findings) or a decrease (two supportive findings).

The increase in hyperactive symptoms can be explained by the restricted opportunities for physical activity during the pandemic due to stay-at-home orders, closures of schools, sport clubs, playgrounds, gyms, public swimming pools, etc., and the cancellation of leisure time activities associated with physical exercise. The lack of evidence indicating an increase in disruptive behaviour problems can be explained by the stability of underlying disorders and dispositions. Furthermore, the display of such symptoms is often situation-specific, meaning symptoms only become evident in certain social settings, for example, in school. Exposure to these situations was reduced during the pandemic due to stay-at-home orders, and school closures potentially led to limited chances to exercise and observe these problematic behaviours.

In line with previous research, the findings show an increase in mental health problems and symptoms of depression and anxiety in children, adolescents, and adults in the context of disasters in general [21, 22, 25, 28] and the COVID-19 pandemic in particular [9, 34, 35, 39–41, 122, 123]. However, due to highly heterogeneous data, the few existing meta-analytic studies regarding mental health effects of the COVID-19 pandemic based on longitudinal data [42–44] indicate no or only slight changes in mental health in the general population. This high variability in findings across studies is also observed in this review, in particular regarding the more global measures of mental health indicators (e.g., internalising symptoms). Negative effects of the pandemic on children’s mental health became more detectable when specific symptom groups were examined separately and when studies were sorted by age of participants. This fits with the assumption that effects might differ across different regions, social groups, and contexts, which we discuss below.

### Changes in mental health during changes in pandemic intensity and restrictions

According to the literature, it can be expected that pandemic-related health protection measures, in particular confinement and quarantine, have a negative effect on child and adolescent mental health [37, 124–126]. Consequently, a reduction in restrictions over the course of the pandemic should be associated with decreasing symptoms of poor mental health. However, not all reviewed studies that assessed the changes in mental health indicators and symptoms over the course of the pandemic support this notion. Nonetheless, more findings indicate a decrease in symptoms after a lift of restrictions (*n* = 9) than no change in symptoms (*n* = 8) or an increase in symptoms (*n* = 2).

This conclusion is in line with data from the newest wave of the study by Ravens-Sieberer et al. [127]. This German study shows that the levels of health-related low quality of life and internalising symptoms in autumn 2021 were higher than pre-pandemic levels. However, health-related quality of life and mental health improved from spring 2020 and winter 2020/21 to autumn 2021. The study authors explain this effect by lower infection rates, higher vaccination rates, and loosening of restrictions.

Reasons for the negative effect of quarantine, confinement, and lockdown on child and adolescent mental health are diverse. For example, Mohler-Kuo et al. [128] found in their survey on stress factors during lockdown in Switzerland that the most common sources of stress were the disruption in social life, the breakdown of normal routines, the cancellation of important plans and events, and the uncertainty and unpredictability of the duration of the pandemic. These were in addition to distressing news coverage, rapidly changing recommendations, and fear of infection and the pandemic itself. Overall, it must be considered that protection measures usually correlate highly with pandemic intensity and infection rates. Contrarily, restrictions are usually lifted when infection rates ease. Thus, a unique contribution of the lifting of containment measures independent from infection rates cannot be assumed [129].

### Changes in mental health related to individual risk and protective factors

Overall, the reviewed studies suggest that low socio-economic status, financial worries, material hardship, lack of space, negative home-schooling experience, bad physical health, and diagnosis of a neurodevelopmental disorder are the key risk factors for experiencing stronger negative mental health effects due to the pandemic (see Table 5). There is also evidence that children and adolescents reported to have experienced constant high levels of mental health problems before and during the pandemic (due to early childhood stress, maltreatment experiences, certain chronic mental health problems, special needs, and socio-economic disadvantage) were less likely than their healthy peers to display an increase in mental health problems in response to the onset of the pandemic [63, 77, 86, 88]. Their mental health was also mostly unrelated to the changing infection rates and health protection measures. This means that they did not benefit from the lifting of restrictions or the easing of the pandemic situation as their same-aged peers did. This is a rather unexpected finding in light of previous research that has proposed that factors such as prior traumatic experiences or dependency on special psychological support enhance the risk of experiencing negative mental health effects in the face of a disaster [21, 27, 41].

We further investigated age effects by grouping the reviewed studies by participant age (see Table 4). Most evidence for an increase in mental health problems (59 supportive vs. 50 non-supportive findings) and a decrease in indicators of good mental health (14 supportive findings vs. four non-supportive findings) was reported for the adolescent age group (i.e., individuals approximately 12–18 years of age). Within this age group we found strong and convincing support for an increase in depressive (17 supportive findings vs.  nine non-supportive findings) and anxiety symptoms (13 supportive vs. seven non-supportive findings), and a decrease in quality of life and well-being (eight supportive findings vs. two non-supportive findings).

A general trend of increasing mental health problems also appeared in school-aged children (i.e., individuals approximately six–12 years of age) with 37 studies reporting an increase in indicators of mental health problems, 22 not finding a change in symptoms, and 17 reporting a decrease in mental health problems. In contrast to adolescents, we found no clear evidence for an increase in depressive and anxiety symptoms in this younger age group.

Hence, adolescents might have suffered more strongly from reduced contact with peers and from heightened demands for personal responsibility (e.g., self-directed learning). Additionally, literature on the psychological development in childhood and adolescence states that adolescents are more vulnerable to social stressors, such as isolation and loneliness [130] and that symptoms of affective disorders rise significantly in adolescence [131, 132]. This age-related difference might also be partially due to the increasing introspective ability of adolescents that allows them to report more reliably than do children on internalising symptoms. Another reason might be that parent-reports, typically associated with an underreporting of internalising symptoms, were used for children but not for adolescents.

For younger children (i.e., individuals approximately under six years of age), most studies do not indicate a change in mental health problems (11 supportive vs. eight non-supportive findings). However, this age group is severely under-investigated. Moreover, the reliability and validity of results might be limited due to difficulties in assessing symptoms in such young children and the reliance on parent- or educator-reports in this review. Therefore, it is not possible to draw clear conclusions for this age group, though it seems that it is less affected by negative mental health effects of the pandemic than are older age groups.

Gender effects were not consistently investigated and reported in the reviewed studies. Of the 14 studies finding and reporting a significant gender effect, 11 studies identified female gender as a risk factor for higher levels and/or a stronger increase in certain indicators of poor mental health and five studies identified male gender as such a risk factor (see Table 5). Females were reported to be at a higher risk for higher levels and/or stronger increases in internalising, anxiety and depressive symptoms, stress, and lower levels of well-being than males. At the same time, males seemed more prone to attention problems, addictive gameplay, and sharper decreases in quality of life and life satisfaction than females. These gender differences were nearly exclusively found in adolescent samples.

Regarding socio-economic variables, children and adolescents who grow up with a single parent and those who do not have siblings were particularly at risk to show decreased levels of mental health during the pandemic. Further, housing situations that provided only limited living space and no access to green spaces were associated with greater increases in and/or levels of mental distress. A low socio-economic status and family financial worries were also identified as important risk factors, especially if the children did not already experience heightened psychological strain prior to the pandemic.

Other individual factors that acted as risk factors for mental health include poor physical health prior to the pandemic, diagnosis of a neurodevelopmental disorder, and dysfunctional or lacking coping and emotion regulation strategies. Parental strain and psychological strain, particularly in the form of parental symptoms of anxiety, depression, and substance abuse were found to be important influential factors for child and adolescent mental health during the pandemic. Further risk factors for poor mental health in the family environment include negative parental coping strategies, dysfunctional parenting styles, and overall family stress and instability.

Pandemic-specific factors that led to more negative mental health effects were the perceived lifestyle impact of the pandemic-related policies on the one hand, and stress due to the pandemic situation on the other. The former includes negative changes in the parental job situation, the experience of isolation and loneliness, frustration and boredom, and negative experiences during home-schooling. The latter comprises fears of oneself or family and friends becoming infected with the virus, general health-related concerns, and worries due to the uncertainty of the pandemic situation.

Behavioural factors reported to contribute to negative mental health outcomes were prolonged screen time, less physical exercise, disrupted sleep patterns, and staying mainly inside during times of confinement. Factors reported to promote mental health or mitigate negative mental health effects include access to green spaces and time spent in nature during times of confinement. Good physical health and health-related behaviours such as regular physical exercise, a healthy diet, structured routines, and regular sleep patterns reportedly helped protect child and adolescent mental health. Children’s and adolescents’ positive and adaptive coping mechanisms and emotion regulation abilities and social support through families and friends have also been identified as protective factors. Family functioning and a positive family climate have been found to help reduce negative mental health effects.

While many of the risk factors named are hardly modifiable (e.g., socio-economic variables, chronic mental and physical health conditions, parental mental health and parenting abilities, pandemic-related stressors), it is important to point out that most of the described protective factors are behaviour-based and can be adjusted (e.g., regular physical exercise, regular sleep, contact with friends, spending time in nature). Consequently, even though there might be unpreventable factors that heighten the risk for negative mental health consequences, there are also multiple viable preventive measures that can foster positive coping and protect mental health.

### Reasons for inconsistencies in findings

The variability in the study samples, for example, times and locations (i.e., country/region) of assessments, might be a strong factor in the inconsistency of findings among the reviewed studies. Differing phases of the pandemic and locations are related to variations in infection rates and health protection measures and to the duration and intensity of exposure to the pandemic at the time of assessment.

The timing of the assessments is particularly important in studies that investigated changes in symptoms over the course of the pandemic. An explanation for the fact that some studies did not find a decrease in symptoms after a lift of restrictions might be that recovery takes time and might not be immediately visible in the assessments. Some stressors named above, such as unpredictability of the situation and fear of infection, have most likely continued to be a burden on mental health even with lifted restrictions.

Additional influencing factors leading to higher heterogeneity in findings might be differences in sample characteristics (e.g., age, socio-economic status), methodological approaches (over 60 different assessment tools are represented), conceptualisation of mental health problems, and definition and operationalisation of outcome variables. An important reason for the heterogeneous findings on the effects of health protection measures might be that mental health is not only influenced by the severity of protection measures but also associated with other characteristics of the pandemic (e.g., infection rates, death tolls). It is difficult to distinguish the effects of the intensity of protection measures from those of the severity of infection rates and the perceived pandemic threat. For example, one of the reviewed studies compared mental health effects among three European countries and showed a greater increase in mental health problems in the countries with greater restrictions, which also had higher infection and death rates at the time [60].

In fact, it has been proven that both policy stringency and pandemic intensity affect mental health to a similar degree [129]. This means that minimising transmission of the virus and death rates by potentially stricter health protection measures might indeed be protective against negative mental health effects due to the pandemic’s intensity. It also seems more appropriate to use separate measures for distinct symptom categories (e.g., depressive symptoms, psychosomatic symptoms) than global measures (e.g., internalising symptoms) because the latter might be less suitable for accurately capturing a change in single symptoms. The same explanation can be applied to the heterogeneous findings for complex symptom groups (e.g., anxiety) that comprise many different forms of a syndrome (e.g., generalised anxiety, health-related anxiety, school-related anxiety) that might have developed differently during the pandemic (see e.g., 119). Finally, there is evidence for age-related differences in the change of mental health symptoms due to the pandemic. For instance, it seems that adolescents have experienced an increase in anxiety symptoms while children have not. Looking at these different effects altogether in the total study sample of the scoping review might have contributed to the heterogeneity of our findings.

### Strengths and limitations of the current review

To our knowledge our review is the first to include only studies with multiple assessment waves, which allows for the detection of changes in mental health that can be clearly attributed to the COVID-19-pandemic. This is a huge advantage over previous papers that mainly relied on cross-sectional estimations of the prevalence of certain mental health indicators. Furthermore, this review goes beyond studies conducted in the early months to include research that assessed mental health over the first one and a half years of the pandemic. The large sample size of our study and inclusion of data from 21 countries on 4 continents allow for a higher generalisability of results. Conclusions drawn from this work are, thus, not restricted to the initial outbreak and beginning of the pandemic but are suited to estimate broader and more long-term effects.

There is potential for errors in the study selection because the identified studies were primarily assessed for eligibility, selected, and synthesised by only one researcher. Another reason for a possible distortion in the data might be the effects of publication bias due to, for example, the under-publication of non-significant results (the file drawer problem; see, e.g., [133, 134]). This review includes published and peer-reviewed articles only. We did not attempt to search for unpublished articles to ensure the scientific quality of the studies. There is a chance that the results reviewed here, therefore, represent a biased sample of relevant data. Because we did not statistically assess the size of the publication and reporting bias, we cannot estimate effects these biases may have on our results.

The conclusions drawn in this paper are most representative of western, educated, industrialised, rich, and democratic societies (WEIRD-bias, c.f., [135]) because most studies were conducted in North American and Western European countries. In the face of a global pandemic that has most likely affected socially and economically disadvantaged groups the most, this is a serious limitation to this review.

Most of the studies in the sample concentrated on the first months of the pandemic, March to June 2020. Furthermore, two-thirds of all included studies were conducted during stricter confinement rules. The lack of studies conducted both after summer 2020 and outside heightened restrictions means that the long-term effects of the pandemic situation, which has lasted much longer the initial period, are not completely evaluable.

The sample is most representative of early adolescent years and does not allow for clear statements about effects on younger age groups, especially early childhood.

A major limitation of this scoping literature review is that all included studies rely only on assessments of mental health via child or adolescent self-, caretaker-, or teacher-reports on questionnaires, which were presented online in most cases. None of the included studies used clinical evaluations by mental health professionals (e.g., diagnostic interviews), which would be necessary to form solid clinical diagnoses [136]. Moreover, the lack of studies using multiple informants to report on a child’s or adolescent’s mental health limits the validity of results. Consequently, the trends observed in this study do not allow for conclusions about the change of the prevalence of diagnoses but are rather suited to estimate changes in the prevalence of mostly self- or parent-reported symptoms.

We neither assessed nor accounted for the methodological quality of the reviewed studies. We also did not analyse the effect sizes of findings. Additionally, it is nearly impossible to distinguish the extent to which the reported effects were caused by the pandemic itself or by certain pandemic-related stressors, health protection measures, or methodological issues. This reduces the options for summarising the findings to the qualitative method of vote-counting, which is substantially inferior to quantitative statistical methods of synthesising research findings. Hence, the purpose of this paper is an accumulation and summary of the research currently available and an indication of general directions of changes in child and adolescent mental health due to the COVID-19 pandemic.

### Implications for intervention strategies to meet the mental health needs of children and adolescents

This paper shows that children’s and adolescents’ mental health problems and symptoms of mental health disorders increased from before to during the pandemic. Missed chances and milestones of development and the delay in diagnosis and treatment will have long-term mental health consequences. It is obvious, therefore, that the need for psychological care for children and adolescents has risen and is still rising worldwide. The situation of mental health care was already precarious before the pandemic. It has now become even more painfully evident that there is a great dissonance between demand and availability of mental health care in many places worldwide. Therefore, it is important to invest in research, creation, and implementation of intervention strategies to react to this growing need for psychological care. Furthermore, this review has shown that it is important to balance health protection and infection control measures with child and adolescent protection and guarantee of societal participation.

In general, continuity and stability of access to school, mental and physical health care, and social services are essential for children’s and adolescents’ mental health. Further disruptions should be avoided at all costs. Decisions about the implementation of certain interventions should always be based on expertise and empirical research to allow for efficiency in both the prevention of infections and protection of children’s and adolescents’ mental well-being. Some concrete measures that could help to reach this goal are proposed in the following.

It is important to facilitate access to mental and physical health care and to social and community services to support children, adolescents, parents, and families in need. The pandemic has shown that there is oftentimes room for improvement in the online resources of such services, which can be especially helpful in cases where more physical distancing is required, for instance, in times of increasing infection rates or for individuals with physical health risks. Easily and quickly accessible psychosocial support services, such as cost-free helplines, can also promptly provide care to individuals in need even when in-person services should be less accessible.

It is crucial to prepare safe day-care and school environments for children and adolescents, for example, by providing air filters in classrooms and creating concepts for health protection in these institutions. These concepts could include recommendations for group and class sizes, personal hygiene measures, or regulations for mask wearing, vaccination, and testing. These measures are not only necessary to protect child and adolescent physical health but also to protect the health of teachers, thereby preventing cancellation of lessons and further strain on the education system.

Improved digital schooling is necessary to prepare for the worsening of the pandemic situation and for future emergency situations. This could also help children who have to stay home due to an infection to not lose touch with school. This measure includes, for example, extension of online structures, provision of online materials, and training in media competence for both teachers and students. Additionally, education authorities must ensure that all students possess the necessary tools and services at home to participate in online schooling (e.g., stable Internet connection, electronic devices).

Even if structures for online schooling are improved, it is important to provide concepts to support vulnerable families by, for example, guaranteeing access to in-person schooling or day-care for children and adolescents in these families. Special attention should be directed at vulnerable families with limited economic and social resources because these factors put not only children but also their parents at higher risk for experiencing psychological distress. Therefore, social services and structures are needed to reliably identify families in need and support them in receiving the help they need (e.g., parenting classes, financial aids, access to day-care).

It is known that the worldwide school closures have led to a loss of education so teachers should consider the deficits associated with home-schooling and school closures when it comes to evaluations, grading, and exams. Further, teacher sensitivity is needed to identify children and adolescents that might have particularly suffered due to the pandemic situation and connect them to support services. Another important task for both educators and parents is to provide children and adolescents with accurate information about the current pandemic situation and necessary health protection guidelines in age-appropriate ways. This can help children and adolescents understand the current situation, give them room to voice their worries and fears, and prevent the spread of misinformation and panic.

In the family context, it is not only important to consider child and adolescent mental health but also the mental health of parents who have also suffered from the pandemic situation. It is known that parent psychopathology is closely linked to their parenting abilities and children’s psychological distress [137]. Healthy, empathic, and friendly family interaction, communication, and parenting competence should be promoted and fostered by, for example, providing access to parenting programmes or family counselling [138]. Positive parent–child relationships, the presence of secure attachment figures, good caregiving, parenting that encourages emotional expression, acceptance, and positive reframing have been reported to protect child and adolescent mental health after exposure to a disaster [23].

Families should also be encouraged to establish structured routines that include time spent outside, physical activity, and regular sleep times, especially in times of home confinement or quarantine. It can be helpful to prepare programmes to animate children and adolescents to engage in physical exercise at home [137]. These approaches can help to counteract the negative effects of quarantine or home confinement on mental and physical health. Last, effects of loneliness and social isolation can be prevented by encouraging children and adolescents to stay in contact with same-aged peers by providing access to safe and age-appropriate ways of online communication [137]. Parents should respectfully monitor their children’s online activities to protect them from the dangers of the online world.

### Implications for further research

The current study makes evident that there is need for more longitudinal or repeated cross-sectional studies that investigate the changes in certain symptom categories, because we did not identify enough studies to draw cohesive conclusions about their development. These categories include psychosomatic, post-traumatic stress, and compulsive symptoms, symptoms of substance abuse and addiction and symptoms of behavioural addiction to electronic media.

Our focus on the COVID-19-pandemic’s mental health effects on community samples of children and adolescents helps the generalisability of findings, however, it does not allow for conclusions about the pandemic’s effects on special vulnerable groups. These groups include children belonging to marginalised ethnic, religious, or social groups, children of frontline workers, children and adolescents growing up in low-income families, and children and adolescents suffering from chronic physical or mental health conditions, such as neurodevelopmental disorders. There is evidence that these groups of children and adolescents have been more strongly affected by the pandemic’s effects than the general population [138–141].

Lastly, further research into the COVID-19 pandemic’s mental health effects and into strategies for the mitigation of negative outcomes is important far beyond the current health crisis. There is a causal link between climate change and an increase in the frequency of pandemic outbreaks (e.g., [142, 143]). This means that if we fail to appropriately react and fight against the current climate crisis, we will have to prepare ourselves for future pandemics. Therefore, it is important and urgent to analyse the effects of the current pandemic in depth and to plan and prepare future actions accordingly based on facts and empirical evidence.

## Conclusion

This scoping review of 69 longitudinal and repeated cross-sectional studies has demonstrated that mental health problems in children and adolescents have increased globally in the first 17 months of the COVID-19 pandemic compared to pre-pandemic data. We have shown that the mental health problems of children and adolescents are positively associated with both the intensity of the pandemic situation (e.g., infection rates, death tolls) and the severity of protection measures, such as confinement and quarantine. Among many other factors we have identified female gender, adolescent age, socio-economic disadvantage, parental psychopathology, dysfunctional family environment, social isolation and loneliness, loss of routines and structure, and the experience of distressing emotions due to health-related worries and uncertainty of the pandemic situation as increasing the chances of suffering negative mental health consequences.

Future research with assessments of the long-term effects of pandemic exposure and of the changes in clinical diagnoses is needed to extend the current findings. This is necessary to more concretely estimate the increased need for child and adolescent mental health care. The protection of children’s and adolescents’ mental health must be prioritised at all costs in a crisis like the COVID-19 pandemic to prevent life-long harm to future generations.

### Supplementary Information

Below is the link to the electronic supplementary material.

Supplementary file1 (DOCX 27 KB)

Supplementary file2 (DOCX 22 KB)

Supplementary file3 (DOCX 32 KB)

## Data Availability

All relevant data are within the manuscript and its supporting information files. This paper was not registered and no protocol was prepared.
