# Supplementary material for: Scoping review: longitudinal effects of the COVID-19 pandemic on child and adolescent mental health
Source: Eur Child Adolesc Psychiatry. 2023 Apr 21;33(5):1257–312. doi: 10.1007/s00787-023-02206-8 (PMC10119016; doi:10.1007/s00787-023-02206-8)
Supplement: Supplementary file 1 — Supplementary file1 (DOCX 27 KB) [file 787_2023_2206_MOESM1_ESM.docx]

**S1 Table. Number of Studies with Data Collection Conducted in a Respective Month in the COVID-19 Pandemic.**

| Months | Feb 20 | Mar 20 | Apr 20 | May 20 | Jun 20 | Jul 20 | Aug 20 | Sep 20 | Oct 20 | Nov 20 | Dec 20 | Jan 20 | Feb 20 | Mar 20 | Apr 20 | May 20 | Jun 20 |
| --- | --- | --- | --- | --- | --- | --- | --- | --- | --- | --- | --- | --- | --- | --- | --- | --- | --- |
| Number of studies | 1 | 16 | 33 | 37 | 29 | 18 | 11 | 8 | 11 | 13 | 11 | 7 | 4 | 2 | 1 | 1 | 1 |
